# Pharmacogenomics of aromatase inhibitors in postmenopausal breast cancer and additional mechanisms of anastrozole action

**DOI:** 10.1172/jci.insight.137571

**Published:** 2020-08-20

**Authors:** Junmei Cairns, James N. Ingle, Tanda M. Dudenkov, Krishna R. Kalari, Erin E. Carlson, Jie Na, Aman U. Buzdar, Mark E. Robson, Matthew J. Ellis, Paul E. Goss, Lois E. Shepherd, Barbara Goodnature, Matthew P. Goetz, Richard M. Weinshilboum, Hu Li, Mehrab Ghanat Bari, Liewei Wang

**Affiliations:** 1 Department of Molecular Pharmacology and Experimental Therapeutics,; 2 Division of Medical Oncology, and; 3 Department of Health Sciences Research, Mayo Clinic, Rochester, Minnesota, USA.; 4 The University of Texas MD Anderson Cancer Center, Houston, Texas, USA.; 5 Memorial Sloan Kettering Cancer Center, New York, New York, USA.; 6 Baylor Cancer Center, Houston, Texas, USA.; 7 Massachusetts General Hospital, Boston, Massachusetts, USA.; 8 NCIC Clinical Trials Group, Kingston, Ontario, Canada.; 9 Patient advocate, Mayo Clinic Breast Cancer Specialized Program of Research Excellence, Rochester, Minnesota, USA.

**Keywords:** Oncology, Therapeutics, Breast cancer, Pharmacogenetics

## Abstract

Aromatase inhibitors (AIs) reduce breast cancer recurrence and prolong survival, but up to 30% of patients exhibit recurrence. Using a genome-wide association study of patients entered on MA.27, a phase III randomized trial of anastrozole versus exemestane, we identified a single nucleotide polymorphism (SNP) in CUB And Sushi multiple domains 1 (*CSMD1)* associated with breast cancer–free interval, with the variant allele associated with fewer distant recurrences. Mechanistically, CSMD1 regulates *CYP19* expression in an SNP- and drug-dependent fashion, and this regulation is different among 3 AIs: anastrozole, exemestane, and letrozole. Overexpression of CSMD1 sensitized AI-resistant cells to anastrozole but not to the other 2 AIs. The SNP in CSMD1 that was associated with increased CSMD1 and CYP19 expression levels increased anastrozole sensitivity, but not letrozole or exemestane sensitivity. Anastrozole degrades estrogen receptor α (ERα), especially in the presence of estradiol (E2). ER^+^ breast cancer organoids and AI- or fulvestrant-resistant breast cancer cells were more sensitive to anastrozole plus E2 than to AI alone. Our findings suggest that the *CSMD1* SNP might help to predict AI response, and anastrozole plus E2 serves as a potential new therapeutic strategy for patients with AI- or fulvestrant-resistant breast cancers.

## Abstract 2

A germline variation within the CSMD1 gene predicts aromatase inhibitor response in breast cancer.

## Graphical abstract

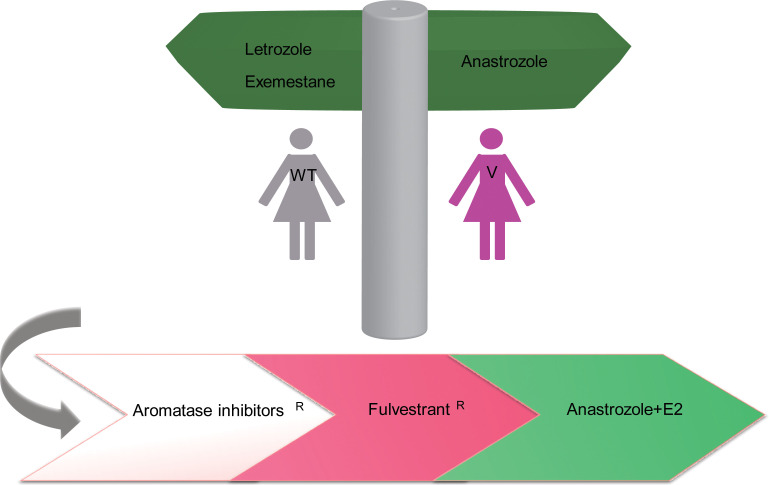

## Introduction

About 70% of primary breast cancers express estrogen receptor α (ERα). Adjuvant endocrine therapy, including aromatase inhibitors (AIs), is a standard treatment for these patients, regardless of tumor size or nodal status. For postmenopausal women with primary ER^+^ breast cancer, AIs are the standard-of-care to prevent relapse and prolong survival (1, 2). In advanced disease, AI-based therapy is a standard initial treatment. Despite their efficacy, about 19% of patients with early-stage disease suffer a recurrence by 10 years (1), and resistance to AIs in advanced or metastatic tumors invariably occurs (3). Therefore, there is a great need to understand the underlying mechanisms associated with AI response and resistance.

There are well-accepted biomarkers associated with both de novo and acquired resistance to endocrine therapy (4, 5), and both host (germline) and somatic (tumor) alterations are known to contribute to resistance (6, 7). For example, the tumor genome has revealed several mechanisms of acquired AI resistance (8–10) such as amplifications or mutations that can activate ESR1 (10) and ligand-independent ERα activation of downstream pathways such as the phosphatidylinositol 3 kinase (11). At the same time, germline single nucleotide polymorphisms (SNPs) in *CYP19A1* have been associated with variation in circulating estrogen concentrations and with breast cancer risk (12).

In postmenopausal women, estrogen is mainly synthesized in peripheral tissues through the action of aromatase (CYP19A1). The main substrates, testosterone and androstenedione, are transformed by aromatase into 17β-estradiol (E2) and estrone (E1), respectively. AIs potently inhibit aromatase and decrease estrogen levels. Two classes of AIs are currently in clinical use: steroidal — e.g., exemestane, which binds aromatase irreversibly — and nonsteroidal — e.g., anastrozole and letrozole, which block the enzyme reversibly (13). While high BMI has been associated with AI resistance relative to tamoxifen (14, 15) — suggesting that variability in estrogen suppression might be associated with clinical outcome — and there are no biomarkers unique to an individual AI, large phase III adjuvant clinical trials do not indicate any difference in efficacy between the 3 AIs (16, 17).

In the present work, following our previous observation that suppression of estrogens is associated with anastrozole treatment outcomes (18), we performed genome-wide association studies (GWAS) using changes in estrogen levels (before and after anastrozole) as the phenotype of interest using patients prospectively enrolled in the Mayo/M.D. Anderson/Memorial Sloan Kettering (designated M3) pharmacogenomics study (19). Here, we identified a series of SNPs that met genome-wide significance. To further determine whether these SNPs in genes associated with estrogen suppression were also associated with long-term clinical outcome, we identified all SNPs within these gene regions based on the 1000 Genome Project and then examined their associations with breast cancer outcomes using GWAS data (7) previously obtained from the Canadian Cancer Trials Group MA.27 trial (16). MA.27 was a phase III trial comparing adjuvant anastrozole and exemestane treatment of postmenopausal women with ER^+^ breast cancer. The GWAS from the M3 clinical study identified SNPs within the human CUB and Sushi multiple domains 1 (*CSMD1*) gene associated with changes in estrogen levels during anastrozole therapy. An additional SNP in *CSMD1* was also found to be associated with breast cancer–free interval (BCFI) in MA.27. The variant SNP genotype was associated with longer BCFI. Functional studies indicated that the SNP altered the CYP19A1 expression in an anastrozole-dependent fashion through transcription regulation. These findings further confirmed our previous observation (18) that anastrozole is different from exemestane and letrozole. Our previous study has already shown that anastrozole, but not letrozole or exemestane, acts as an ERα ligand and degrader, and in the current study, we further show that the addition of E2 could sensitize cells to anastrozole, even in anastrozole- or fulvestrant-resistant models.

## Results

### GWAS of changes in estrogen levels and BCFI GWAS.

**Figure 1:**
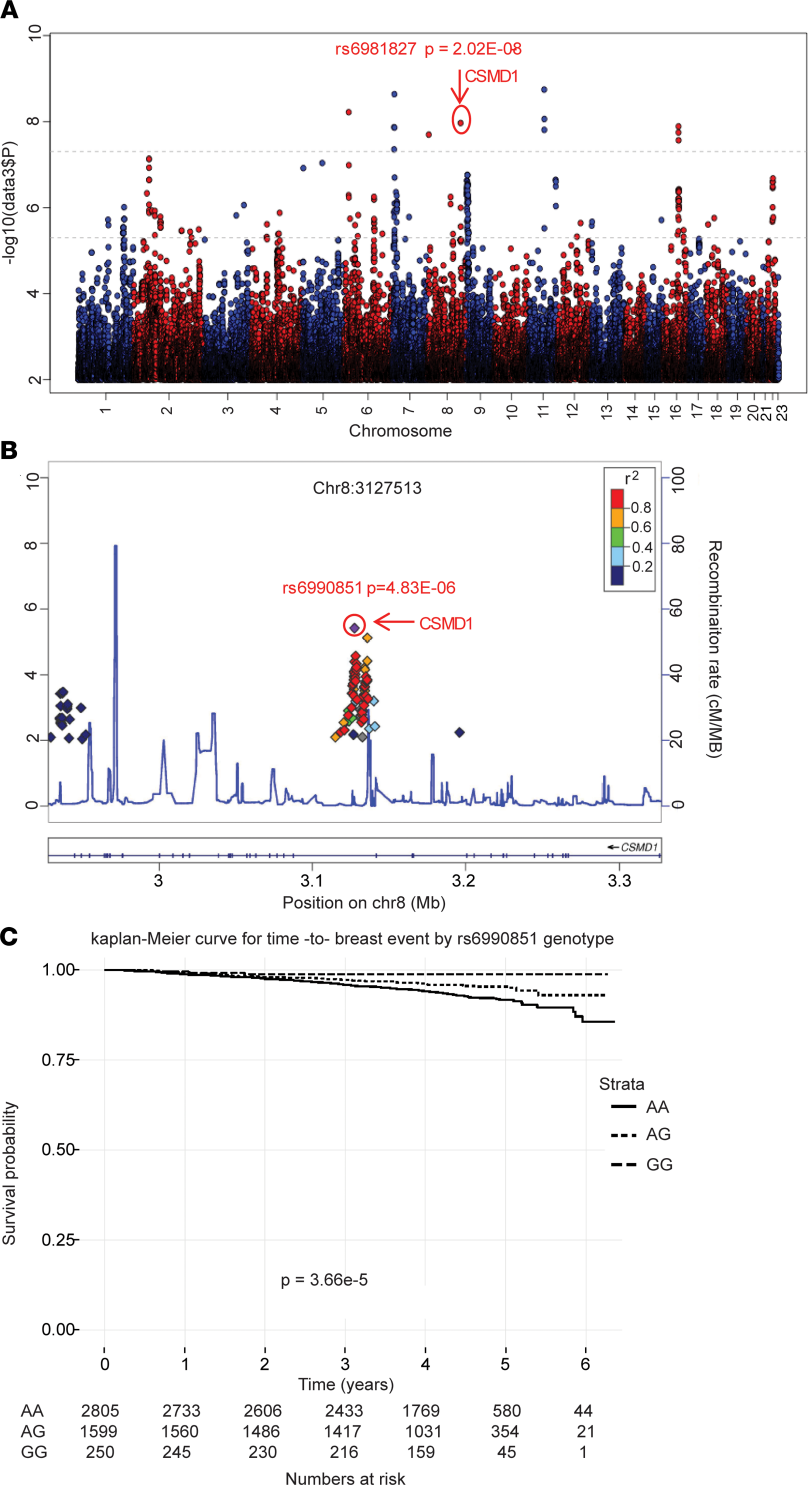
Discovery of *CSMD1* SNPs. (**A**) Manhattan plot of M3 GWAS results for change in estradiol upon anastrozole treatment. (**B**) Genomic position of *CSMD1* SNP associated with BCFI in MA.27. (**C**) Kaplan-Meier plots of time to breast events in MA.27 for different copies of *CSMD1* variant allele rs6990851. *P* value represents stratified Cox-proportional hazards analysis.

To identify SNPs associated with AI estrogen suppression, we performed GWAS for changes in estrogen levels before and after anastrozole in 624 postmenopausal women with resected early-stage ER^+^ breast cancer accrued through the M3 study (19). The flow diagram (Supplemental Figure 1A; supplemental material available online with this article; https://doi.org/10.1172/jci.insight.137571DS1) shows the number of patients included in the analysis. We performed 3 GWA studies for changes in E1, E2, and the sum of E1 and E2 (Figure 1A and Supplemental Figure 1B). Common SNPs reaching genome wide significance (*P* < 5 × 10^–8^) among the 3 GWAS included rs2449598 within *DLG2* on chromosome 11 (*P* = 2.23 × 10^–10^ to 1.24 × 10^–8^), rs1437153 near *CDH11* on chromosome 16 (*P* = 2.82 × 10^–9^ to 2.7 × 10^–8^), and rs6981827 in the intron of the *CSMD1* gene on chromosome 8 (*P* = 2.02 × 10^–8^ to 2.12 × 10^–8^) (Figure 1A; Supplemental Figure 1B; Supplemental Table 1). The variant alleles for these SNPs were associated with less estrogen suppression during AI treatment.

Because M3 was a pharmacokinetic and pharmacodynamic study, outcome data were not available, and we examined the SNPs identified in M3 in the MA.27 trial where we had long-term follow-up (16). We identified all SNPs in *DLG2*, *CDH11*, and *CSMD1* gene regions (100 kb up- and downstream of the gene) using data from the 1000 Genome Project and determined their association with BCFI in MA.27 for which GWAS results were available (7). The most significant association with the MA.27 BCFI phenotype was the rs6990851 SNP in the *CSMD1* gene (*P* = 4.83 × 10^–6^, HR = 0.56) (Figure 1B). The variant allele (minor allele frequency [MAF] = 0.21) for rs6990851 was associated with longer BCFI (Figure 1C). No difference was observed between the 2 drugs with regard to SNP effect on BCFI. The rs6990851 SNP from MA.27 and the rs6981827 from M3 were only weakly linked in the M3 breast cancer patients (*r^2^* = 0.014) and the MA.27 population (*r^2^* = 0.02). Therefore, neither of the SNPs was significantly associated with the other phenotype. The *CSMD1* variant SNP, rs6981827 (MAF = 0.05), associated with less estrogen change in the M3 study (effect size = –0.60), was not an expression quantitative trait loci (eQTL) SNP for *CSMD1* gene expression based on genotype-tissue expression (GTEx), while the *CSMD1* SNP rs6990851, which was associated with longer BCFI, was associated with higher *CSMD1* expression level in adipose and brain tissues, and with a trend in breast tissue (Supplemental Figure 1C).

### Rs6990851 showed SNP- and anastrozole-dependent transcriptional regulation of CSMD1 and CYP19A1.

**Figure 2:**
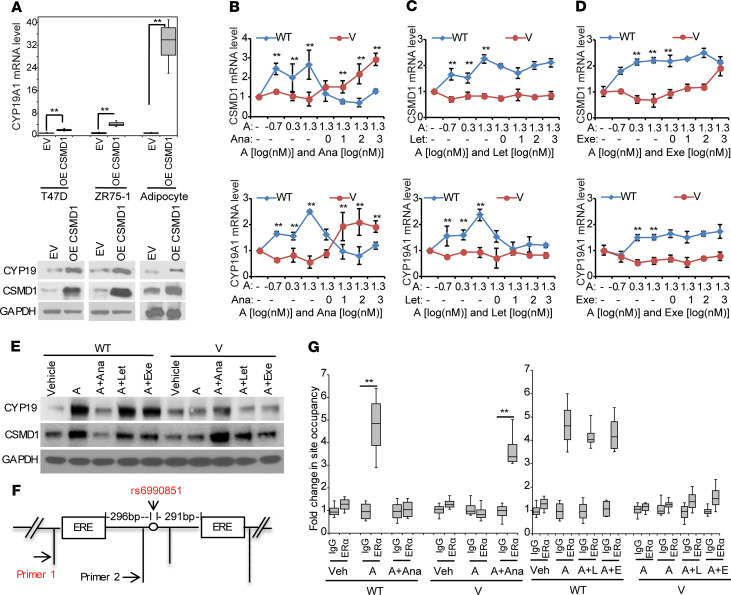
*CSMD1* SNP androstenedione– and SNP anastrozole–dependent regulation of *CSMD1* and *CYP19A1* expression. (**A**) *CYP19A1* mRNA expression levels in *CSMD1*-overexpressed breast cancer cells and human adipocytes. (**B–E**) SNP-dependent gene regulation of *CSMD1* and *CYP19A1* expression in the presence of increasing concentrations of androstenedione (A) or 20 nM androstenedione plus increasing concentrations of AIs (Ana, anastrozole; Let, letrozole; Exe, exemestane) in lymphoblastoid cell lines (LCLs) selected based on the rs6990851 SNP genotype. *CSMD1* WT, homozygous WT LCLs (*n* = 5); *CSMD1* V, homozygous variant LCLs (*n* = 5). (**F**) Schematic figure shows the EREs surrounding the rs6990851SNP. Primers 1 and 2 were used in the ChIP assays to determine the regions around the 2 EREs. (**G**) ERα ChIP assay shows SNP-dependent ERα binding to the ERE region that is 296 bp upstream from the rs6990851 SNP in LCLs with different genotypes treated with the indicated drugs. Error bars (**A**, **B–D**, and **G**) represent ± SEM of 3 independent experiments. *P < 0.05; **P < 0.01. Two-way ANOVA.

We first determined whether the expression of *CSMD1* might be estrogen dependent. We treated ER-expressing ZR-75-1 and T47D cells with 0.1 nM E2 and observed that *CSMD1* mRNA was significantly induced (*P* < 0.01; Supplemental Figure 2). Because the known mechanism of action of AIs is to block aromatase activity, we then tested a possible relationship between the expression of CSMD1 and CYP19A1. Overexpression of CSMD1 increased CYP19A1 expression levels in breast cancer cells and human adipocytes (Figure 2A). We then used ENCODE data to search for putative estrogen response elements (EREs) and identified 2 EREs within 500 bp of the *CSMD1* SNP, rs6990851. Rs6990851 mapped to intron 1 of *CSMD1*, and 2 EREs were detected at 291 bp upstream and 296 bp downstream, respectively, while rs6981827 did not have any EREs within 500 bp, in addition to its lack of eQTL relationship with *CSMD1*. Therefore, we focused on rs6990851 for further functional studies. Based on our prior experience of studying SNP effects on gene expression and hormonal therapy, we took advantage of cell lines selected on the basis of *CSMD1*genotype from a genomic data–rich panel of lymphoblastoid cell lines (LCLs) that has already proven to be a powerful tool for generating and testing pharmacogenomic hypotheses (20, 21). We first exposed LCLs selected based on the rs6990851 SNP genotype of hormones or AIs to determine the effect of the SNP on *CSMD1* and *CYP19A1* expression. This is a strategy that we commonly used to determine the functional impact of GWAS signals (7, 20). LCLs with homozygous WT or variant genotypes for the *CSMD1* rs6990851 SNP were treated with increasing concentrations of androstenedione, a precursor of estrogen, and *CSMD1* and *CYP19A1* mRNA levels were determined. Cells with homozygous WT SNP genotype showed increased expression of *CSMD1* and, in parallel, *CYP19A1* expression (Figure 2, B–D). In contrast, in LCLs with the variant allele, *CSMD1* and *CYP19A1* expression was virtually unchanged with androstenedione. Importantly, addition of anastrozole reversed the expression patterns of *CSMD1* and *CYP19A1* (Figure 2B). Specifically, addition of anastrozole increased *CSMD1* and *CYP19A1* expression in cells with the variant SNP genotype but decreased *CSMD1* and *CYP19A1* levels to or below baseline in cells with the WT allele (Figure 2, B and E). Interestingly, neither letrozole nor exemestane (Figure 2, C and D) significantly changed the expression patterns of *CSMD1* and *CYP19A1* compared between WT and variant LCLs.

To determine whether the SNP impact on gene expression reflects differential binding of ERα to EREs that are located in close proximity of rs6990851, we performed ChIP assays using an ERα antibody. We found that the rs6990851 SNP effect occurred through the ERE located 296 bp upstream from the SNP (Figure 2F) and resulted in an increased binding of ERα in WT cells treated with androstenedione. A similar result was observed in variant cells when anastrozole was added (Figure 2G, left panel). However, no differential binding to the second ERE located 291 bp downstream from the SNP was detected (Supplemental Figure 3). Furthermore, we did not observe any SNP-dependent binding with letrozole or exemestane (Figure 2G, right panel).

### Rs6990851 alters anastrozole response through CSMD1 and CYP19A1.

**Figure 3:**
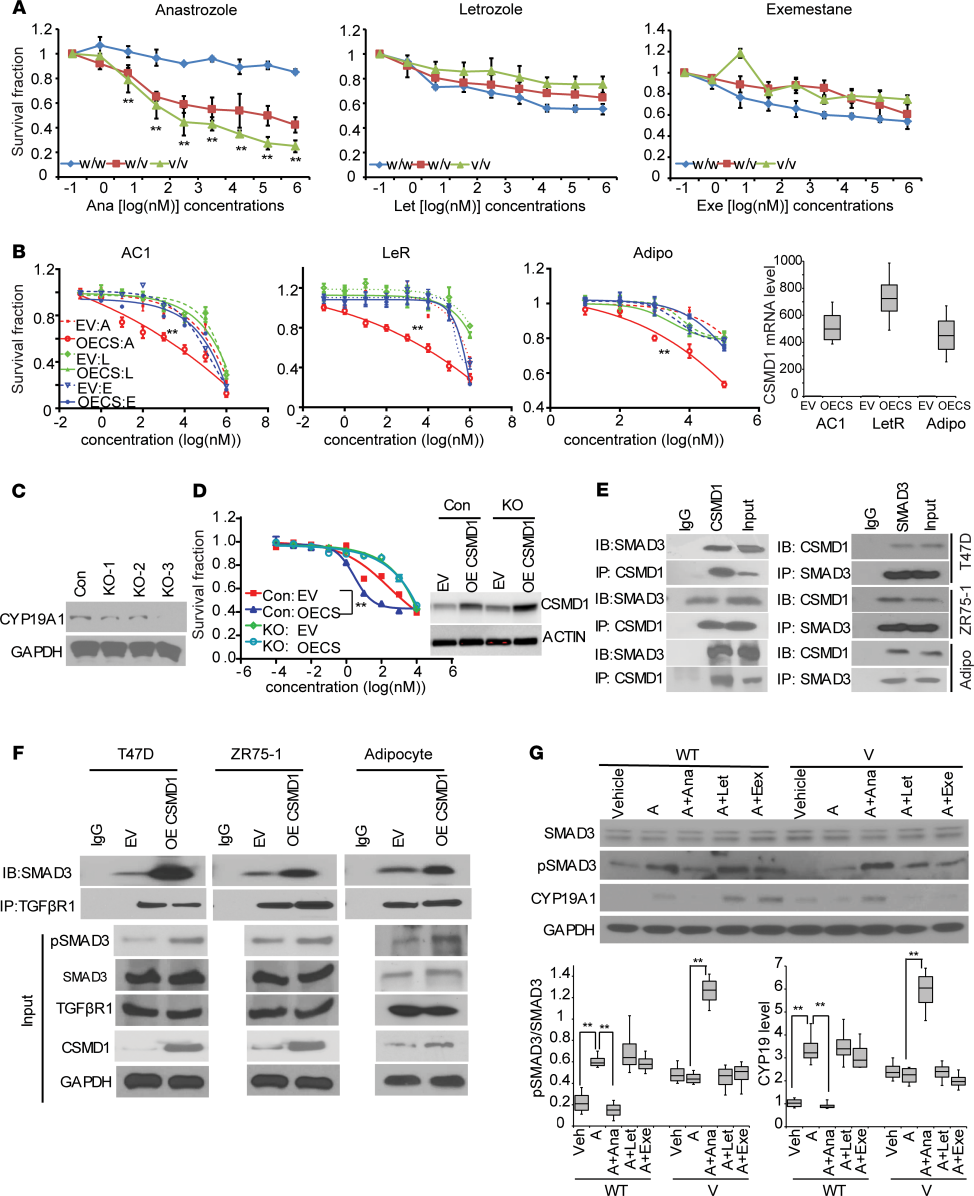
Effects of *CSMD1* SNP on anastrozole response and mechanisms involved in *CSMD1* regulation of *CYP19*. (**A**) *CSMD1* SNP-dependent effect on AI responses. w/w, homozygous WT (*n* = 5); w/v, heterozygous (*n* = 5); and v/v, homozygous variant LCLs (*n* = 5). (w/w versus w/v, *P* < 0.0001; w/w versus v/v, *P* < 0.0001). (**B**) Increased anastrozole sensitivity in cells overexpressing *CSMD1* (empty vector:Ana [EV:Ana] versus OE CSMD1:Ana, *P* < 0.0001). Side panel shows mRNA expression in cell lines tested. MCF7AC1 (AC1), AC1-LetR (LetR), and adipocyte. (**C**) Western blot showing CYP19A1 expression in 3 single clones of *CYP19A1*-KO T47D cells using CRISPR/Cas9. CYP19A1-KO clone 3 (KO-3) was selected for further experiments. (**D**) Anastrozole sensitivity after overexpressing CSMD1 in *CYP19A1*-KO T47D cells (KO) and control cells (Con) shows the reversal of anastrozole sensitivity caused by overexpression of CSMD1. (**E**) Detection of CSMD1 and SMAD3 interaction using immunoprecipitation, followed by Western blot analysis with indicated antibodies. (**F**) Increased SMAD3–TGF-βR binding in *CSMD1*-overexpressed cells. (**G**) *CSMD1* SNP effect on pSMAD3 and CYP19A1 protein levels. A, androstenedione; Ana, anastrozole; Let, letrozole; and Exe, exemestane. Data are represented by ± SEM of 3 independent experiments. **P < 0.01. Two-way ANOVA plus Tukey.

We next sought to determine the functional consequences of the *CSMD1* SNP on response to AIs. LCLs homozygous for the variant SNP, which resulted in high CSMD1 expression, were more sensitive to anastrozole than homozygous WT or heterozygous LCLs (Figure 3A, left panel), whereas these different alleles had little effect on letrozole or exemestane sensitivity (Figure 3A, middle and right panels). To assess the role of *CSMD1* in AI response in breast cancer cells, we used the MCF7/AC1 cell line because of its high expression of the AI target CYP19A1. Additionally, letrozole resistant (LetR) (22), a letrozole-resistant cell line, was used to determine the role of *CSMD1* in AI response in an AI-resistant setting. Finally, we also used human adipocytes because adipose tissue is the predominant source of estrogens in postmenopause women (23). In all 3 lines, overexpression of CSMD1 significantly increased anastrozole sensitivity compared with empty vector, whereas overexpression of CSMD1 showed little effect on letrozole and exemestane sensitivity (Figure 3B). These findings were also observed in 2 other ER^+^ breast cancer cell lines (T47D and ZR-75-1) (Supplemental Figure 4). To further define the relationship between CSMD1 and CYP19A1, we attempted to reverse the AI sensitization effect resulting from CSMD1 overexpression by knocking out endogenous *CYP19A1* using CRISPR/Cas9 methodology in T47D cells (Figure 3C). As shown in Figure 3D, *CYP19*-KO abrogated the effect of CSMD1 overexpression on survival, indicating that *CYP19* is a major target that mediates the effect of *CSMD1* on anastrozole response. CSMD1 was recently shown to interact with SMAD3 in melanoma cells (24). SMAD3, activated by TGF-β3, was required for steroidogenic factor-1 (SF-1) binding to the *CYP19A1* type II promoter (25). We therefore investigated whether CSMD1 might be a component of the SMAD3–TGF-β3 receptor (TGF-βR) complex involved in *CYP19A1* transcription regulation. CSMD1 coprecipitated SMAD3 in breast cancer cells and human adipocytes, and a reciprocal immunoprecipitation further confirmed the interaction (Figure 3E). Overexpression of CSMD1 enhanced the interaction between SMAD3 and TGF-βR and resulted in SMAD3 activation, as evidenced by increased pSMAD3 (Figure 3F). This regulation of *CSMD1* on *CYP19A1* was impaired after the knockdown of *SF-1* or *SMAD3* (Supplemental Figure 5). These results suggest that CSMD1 is a scaffolding protein that brings SMAD3 and TGF-βR together and promotes phosphorylation of SMAD3. To further confirm that the regulation of the *CSMD1* SNP on AI response is through the CSMD1/SMAD3/CYP19A1 axis, we treated LCLs carrying either WT or variant SNP genotypes with androstenedione alone or in combination with an individual AI. As shown in Figure 3G, cells with different rs6990851 SNP genotypes showed striking differences in pSMAD3 and CYP19A1 levels. In particular, WT cells had increased pSMAD3 and CYP19A1 levels after androstenedione treatment, and significantly reduced pSMAD3 and CYP19A1 levels when anastrozole was added. Importantly, addition of letrozole or exemestane did not alter the protein levels in WT cells. In contrast, in the variant cells, androstenedione treatment did not change pSMAD3 and CYP19A1 levels compared with vehicle treatment, while addition of anastrozole, but not letrozole or exemestane, significantly upregulated pSMAD3 and CYP19A1 levels. Therefore, rs6990851 upregulated CSMD1 expression in the presence of anastrozole, but not the other 2 AIs (Figure 2), resulting in SMAD3 activation and increased *CYP19A1* gene expression (Figure 3G), leading to increased sensitivity to anastrozole (Figure 3A).

### Anastrozole potentiates estrogen’s effect on ERα transcription activity and ERα degradation.

**Figure 4:**
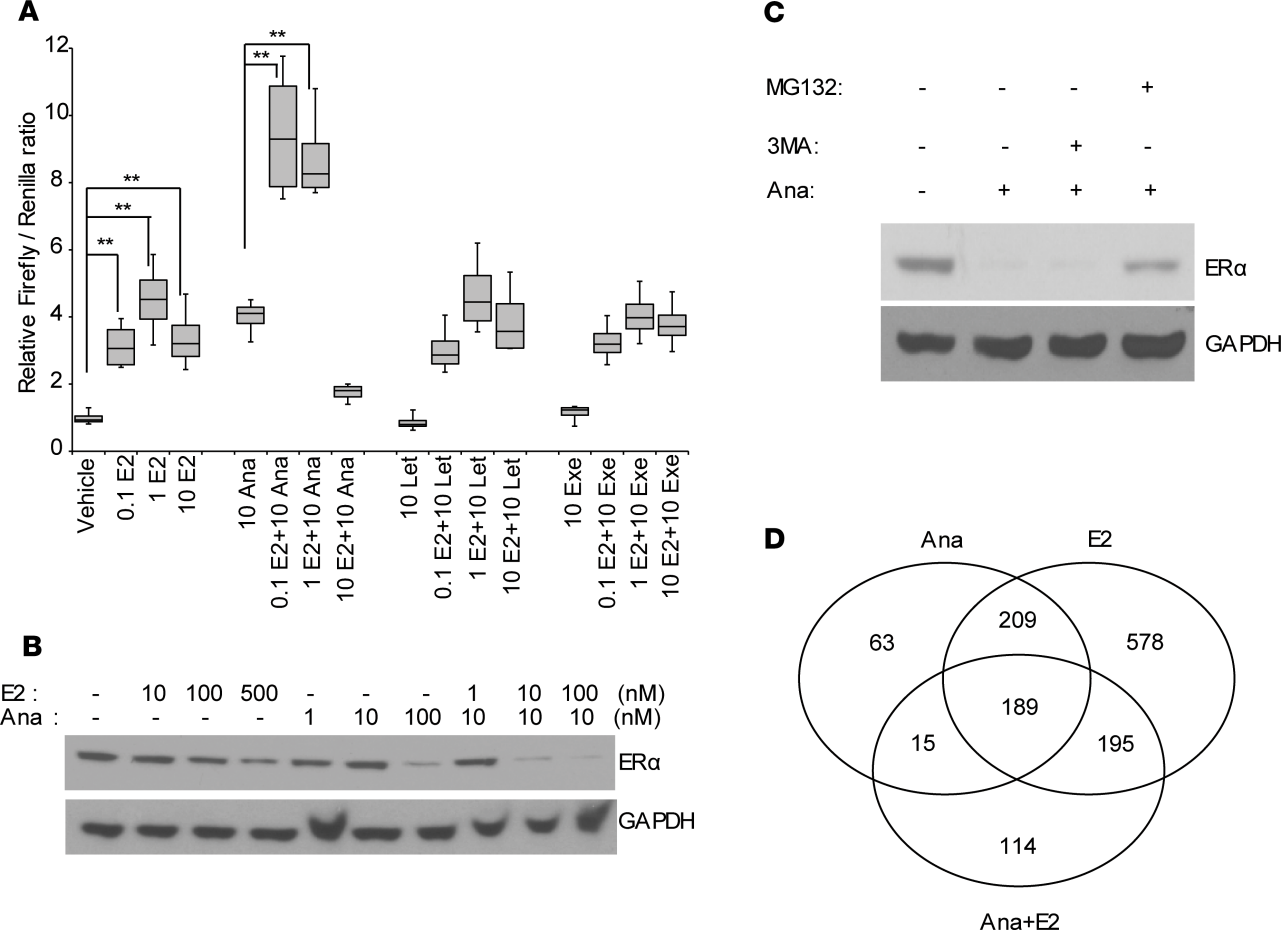
Anastrozole potentiates estrogen effect on ERα transcription activity and ERα degradation. (**A**) ERE-dependent luciferase assay in *CYP19A1* CRISPR-KO T47D cells treated with indicated concentrations of E2, anastrozole (Ana), letrozole (Let), or exemestane (Exe). (**B**) Cells treated with E2 and anastrozole either alone or combined at the indicated concentrations. Western blot was performed to determine ERα protein level. (**C**) Anastrozole induced ERα degradation is proteasome dependent. Cells treated with vehicle, 100 nM anastrozole alone or anastrozole plus 20 μM 3MA, or 10 μM MG132 following Western blot analysis of ERα protein levels. (**D**) Venn diagram showing the overlap between genes differentially expressed in response to anastrozole with or without E2 (FDR < 0.05). Data are represented by ± SEM of 3 independent experiments. **P < 0.01. One-tailed *t* test, Bonferroni correction for multiple testing.

The observation of the *CSMD1* SNP and AI-dependent regulation of gene expression and differential response to individual AI further confirmed our previous finding. Our previous studies indicated that anastrozole is different from the other 2 AIs by possessing a second mechanism of action (18), being an ERα agonist in a similar fashion to E2. It can activate ERα-dependent transcription, but the effect decreased with increasing concentrations of anastrozole due to induction of ERα degradation (18). To further explore the impact of anastrozole effect on ERα activation and degradation, we tested various concentrations of E2 and anastrozole, as well the 2 combined. We showed that anastrozole plus E2 at 0.1 or 1 nM showed approximately 2.4- and 3.01-fold increases, respectively, in luciferase activity compared with anastrozole or E2 alone at that concentration in *CYP19*-KO T47D cells (Figure 4A, P** < 0.001). However, when E2 concentration increased to 10 nM, combination with anastrozole led to a significant inhibition in luciferase activity compared with anastrozole or E2 alone (Figure 4A, P** < 0.001). Letrozole and exemestane had no impact on E2-induced luciferase activity (Figure 4A). These observations suggest that anastrozole has a cumulative effect with low-dose E2 to activate ERα-mediated transcriptional activation. However, at higher concentration of E2, it showed a cumulative effect with anastrozole on ERα degradation, leading to reduced ERE-dependent transcription activity. We further tested the combination effect on ERα protein level. Anastrozole (10 nM) combined with E2 at 10 or 100 nM decreased ERα protein level, while E2 or anastrozole at 10 nM alone was not able to degrade ERα (Figure 4B). Consistent with previous findings (26) showing that high concentrations of E2 led to a proteasome-mediated degradation of ERα protein, we found that the proteasome inhibitor MG132, but not the autophagy inhibitor 3MA, could inhibit the protein degradation induced by anastrozole (Figure 4C).

### Anastrozole regulates a transcriptome distinct from E2 in breast cancer cells.

Since anastrozole can function as an ERα ligand, to investigate whether anastrozole regulates similar or different sets of genes compared with E2, we performed RNA sequencing (RNA-seq) using T47D breast cancer cells treated with anastrozole (10 nM) alone, E2 (0.1 nM) alone or anastrozole (10 nM) plus E2 (0.1 nM). These concentrations were chosen based on the ability of anastrozole to increase luciferase activity without degrading ERα. Anastrozole alone significantly altered levels of 476 transcripts compared with the vehicle control (FDR < 0.05), among which, 398 genes overlapped with E2 single treatment. As shown in the Venn diagram, anastrozole plus E2 treatment resulted in altered expression of 513 transcripts, 204 of which were common with anastrozole alone, and 384 of the 513 were common with E2-alone treatment (Figure 4D, Supplemental Table 2, and Supplemental Figure 6A). Selected genes identified by RNA-seq were further validated by quantitative PCR (qPCR) following anastrozole, E2, or anastrozole plus E2 treatment (Supplemental Figure 6B). Gene set enrichment analysis (GSEA) for anastrozole treatment showed that, compared with E2, anastrozole regulated genes involved in steroid biosynthesis, DNA replication, toxoplasmosis, VEGF signaling pathway, and actin cytoskeleton (Supplemental Table 3). Collectively, these findings suggest that, although at large, anastrozole behaves very similar to E2 with regard to its effect on downstream gene regulation (384 genes), it also preferentially regulates pathways (204 genes) that are different from E2.

### Therapeutic effect of the combination of anastrozole with E2.

**Figure 5:**
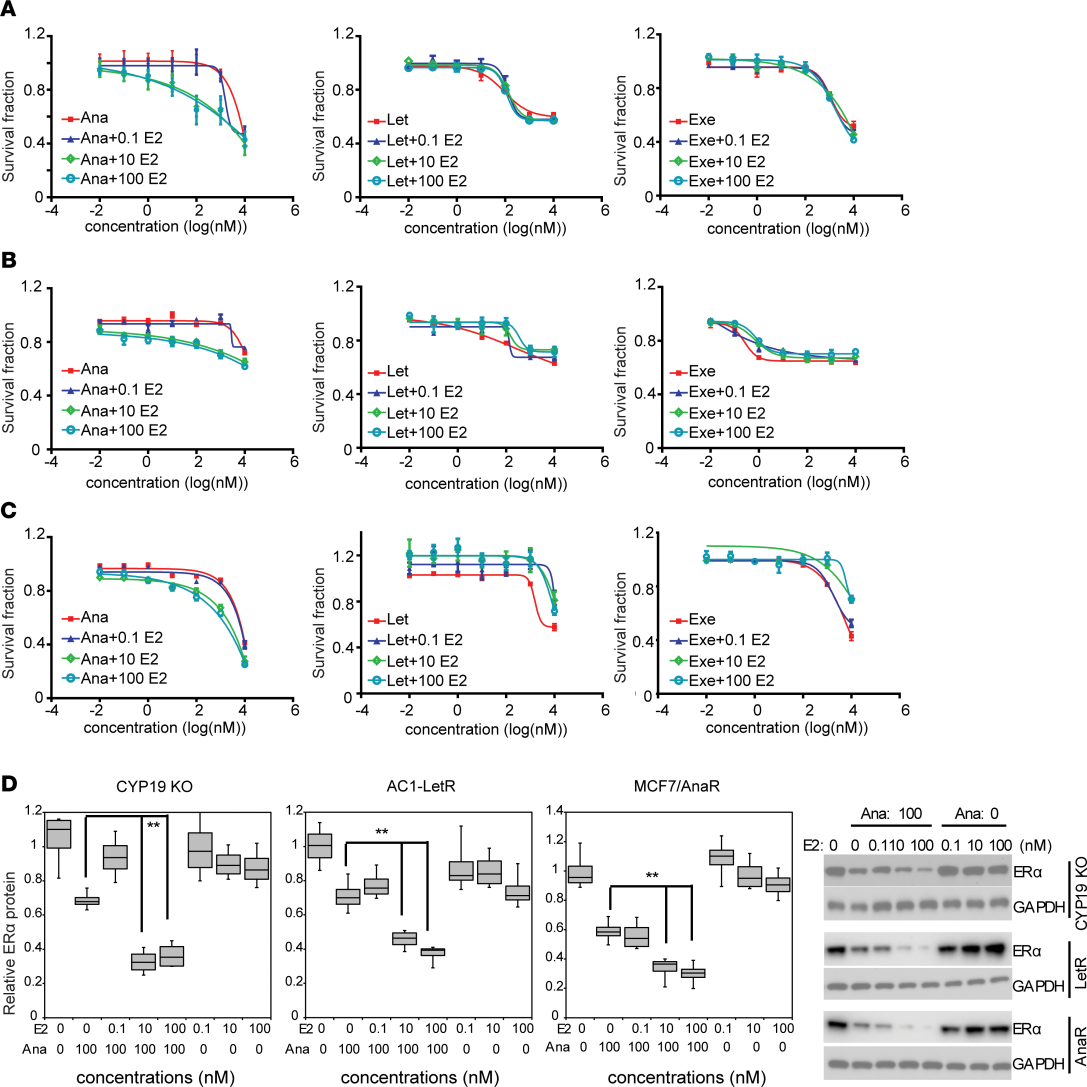
Sensitization effect of E2 on anastrozole response. (**A–C**) Dose response of anastrozole in the presence of E2 at indicated concentrations in CYP19A1-KO cells (**A**) (Ana versus Ana + 10E2, *P* < 0.0001; Ana versus Ana + 100E2, *P* < 0.0001); AC1-LetR cells, the letrozole-resistant cells (**B**) (Ana versus Ana + 10E2, *P* < 0.0001; Ana versus Ana + 100E2, *P* < 0.0001); and MCF7/Ana^R^, the anastrozole-resistant cells (**C**) (Ana versus Ana + 10E2, *P* < 0.0001; Ana versus Ana + 100E2, *P* < 0.0001). (**D**) Western blot and quantification of ERα protein levels after anastrozole and E2 treatment. Data are represented by ± SEM of 3 independent experiments. **P < 0.01. Two-way ANOVA plus Tukey.

**Figure 6:**
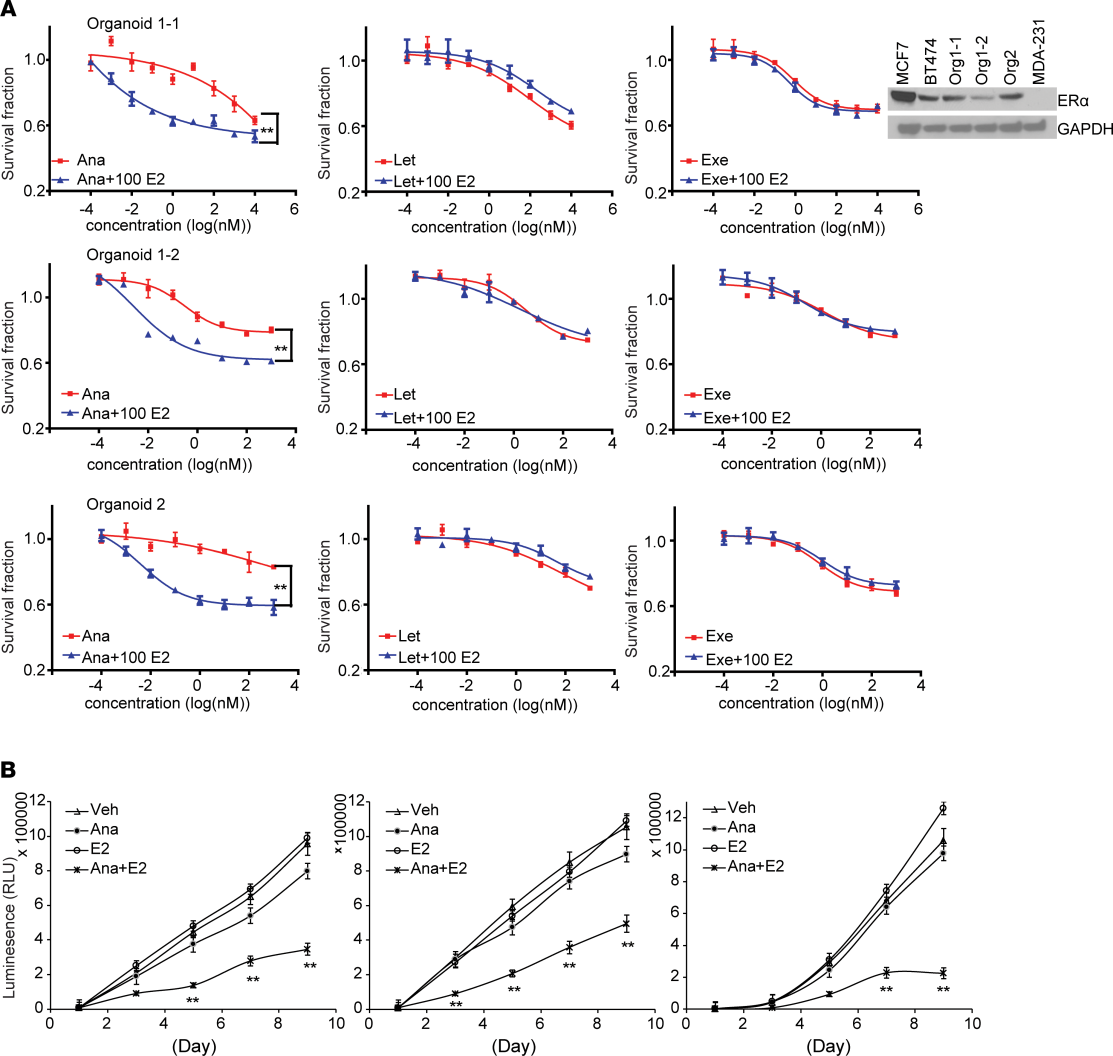
Sensitization effect of E2 on anastrozole response in breast cancer PDX–derived organoids. (**A**) AI dose response in the presence of E2 in 3 organoids derived from breast cancer patients. Western blot showed ER positivity in the organoids (ER-positive control, MCF7 and BT474; ER-negative control, MDA-231). (**B**) Organoid growth curves in the presence of 200 nM anastrozole and/or 100 nM E2. Data are represented by ± SEM of 3 independent experiments. **P < 0.01. Two-way ANOVA plus Tukey.

To test whether the combined effects of anastrozole and E2 on ERα protein degradation might lead to augmented antitumor activity compared with anastrozole alone, we evaluated cytotoxicity in *CYP19*-KO, letrozole-resistant AC1-LetR, and anastrozole-resistant MCF7/anastrozole resistant (Ana^R^) cells (27) with anastrozole with or without E2 treatment. As shown in Figure 5, A–C, 10 and 100 nM of E2 sensitized these cells to anastrozole, but not letrozole or exemestane, and anastrozole plus 10 nM E2 showed better therapeutic effect than any of the 3 AI alone in anastrozole-resistant cells (Supplemental Figure 6C). Treatment with 10 or 100 nM E2 plus 100 nM anastrozole also increased the degradation of ERα protein compared with cells exposed to 100 nM anastrozole alone (Figure 5D). It is important to emphasize that E2 alone, at the concentrations of 10 or 100 nM, did not induce ERα degradation (Figure 5D) — neither did it exert cytotoxic effect (Supplemental Figure 6D). To further confirm the combination effect, we used organoids grown from 2 primary ER^+^ breast cancer patient-derived xenografts (PDXs) (28) and treated with anastrozole plus E2. In all 3 organoids derived from 2 unique breast cancer patients, we confirmed ERα positivity (Figure 5D, Western blot). The dose response after 72-hour exposure to AI alone or in combination with 100 nM E2 was obtained. The number of survival organoids was significantly reduced 72 hours after exposure to anastrozole plus E2, but not to letrozole or exemestane plus E2 (Figure 6A). To further assess the drug combination effects over time (9 days), we used a luminescence survival assay. Here, we selected a 200 nM anastrozole concentration based on the previous organoid response curves (Figure 6A). As shown in Figure 6B, anastrozole and E2 significantly inhibited organoid growth compared with anastrozole alone (*P* < 0.01).

**Figure 7:**
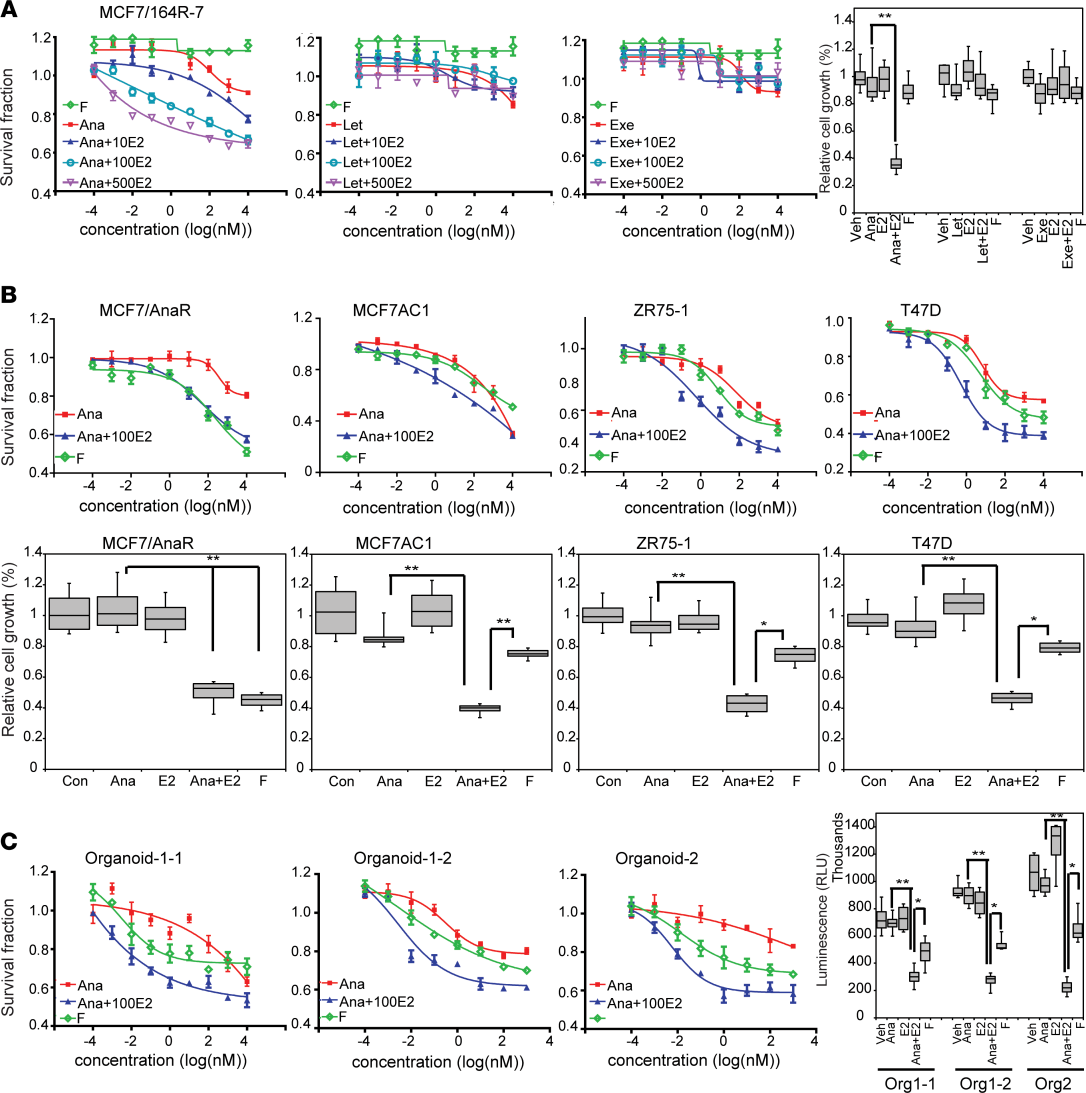
The comparison of efficacy between anastrozole plus E2 versus fulvestrant. (**A**) Effects of anastrozole + E2 in fulvestrant-resistant cells. Fulvestrant (F) versus Ana, *P* = 0.0009; F versus Ana + 10E2, *P* < 0.0001; F versus Ana + 100E2, *P* < 0.0001; F versus Ana + 500E2, *P* < 0.0001; Ana versus Ana + 10E2, *P* = 0.0288; Ana versus Ana + 100E2, *P* < 0.0001; Ana versus Ana + 500E2, *P* < 0.0001. Quantitative analysis of survival cells corrected back to vehicle treatment was performed after 3 days of treatment with indicated drugs, as shown in the bar graph. **P < 0.01. Two-way ANOVA plus Tukey. (**B** and **C**) Comparison of anastrozole + E2 with fulvestrant in anastrozole-resistant MCF7/AnaR and 3 AI-naive cell lines (**B**), as well as in PDX-derived organoids (**C**). Quantitative analysis of survival cells corrected back to vehicle treatment was performed, as shown in the bar graph 3 days after treatment. Data are represented by ± SEM of 3 independent experiments. MCF7/AnaR: F versus Ana, *P* < 0.0001; F versus Ana + 100E2, *P* = 0.2093; Ana versus Ana + 100E2, *P* < 0.0001. MCF7AC1: F versus Ana + 100E2, *P* < 0.0001; Ana versus Ana + 100E2, *P* = 0.0013. ZR-75-1: F versus Ana + 100E2, *P* < 0.0001; Ana versus Ana + 100E2, *P* < 0.0001. T47D: F versus Ana + 100E2, *P* < 0.0001; Ana versus Ana + 100E2, *P* < 0.0001. Organoid-1-1: F versus Ana + 100E2, *P* < 0.0001; Ana versus Ana + 100E2, *P* = 0.0002; Ana versus F, *P* = 0.02. Organoid-1-2: F versus Ana + 100E2, *P* = 0.0013; Ana versus Ana + 100E2, *P* < 0.0001; Ana versus F, *P* = 0.013. Organoid-2: F versus Ana + 100E2, *P* < 0.0001; Ana versus Ana + 100E2, *P* = 0.0002; Ana versus F, *P* = 0.0026. *P < 0.05; **P < 0.01. Two-way ANOVA plus Tukey.

Compared with the third-generation AIs, fulvestrant has superior efficacy and is a preferred treatment option for patients with hormone receptor–positive (HR^+^) locally advanced or metastatic breast cancer (MBC) based on the FALCON trial (29) and in the neoadjuvant setting based on the CARMINA 02 trial (30, 31). However, up to half of ER^+^ MBC patients show intrinsic resistance, and ultimately all of them develop acquired resistance to fulvestrant (29). To test the efficacy of anastrozole plus E2 in the fulvestrant-resistant setting, we treated fulvestrant-resistant MCF7/164R-7 cells (27) with individual AI alone or combined with increasing concentrations of E2. We found that, in fulvestrant-resistant cells, individual AI alone had a slightly better cytotoxic effect than fulvestrant. However, only anastrozole showed an E2 dose-dependent sensitization, and this phenomenon was not observed for letrozole or exemestane (Figure 7A and Supplemental Figure 6E). Since fulvestrant is used in the AI-resistant setting, we then compared the response of anastrozole plus E2 to fulvestrant in MCF7/Ana^R^-2 cells. Our data reveal that the combination of anastrozole with 100 nM E2 showed an effect similar to that of fulvestrant (Figure 7B, top panel). MCF7/Ana^R^-2 cell growth was dramatically reduced in the presence of 100 nM anastrozole plus E2 after 3 days (Figure 7B, bottom panel). Even in endocrine-naive breast cancer cells (MCF7AC1, ZR-75-1, and T47D), anastrozole plus E2 improved the therapeutic effects compared with anastrozole or fulvestrant alone (Figure 7B, top and panels). Finally, anastrozole plus E2 improved the therapeutic response in breast cancer PDX organoids compared with fulvestrant (Figure 7C). Our findings suggest that anastrozole plus E2 could improve outcome in both endocrine naive and -resistant breast cancers.

## Discussion

This study has identified potentially novel common genetic variants associated with estrogen suppression and breast cancer events in women treated with adjuvant AI therapy. None of these SNPs had been previously reported in association studies (7, 12, 32). Our results not only help identify potential new biomarkers for the selection of patients who might benefit from AIs therapy, but also enhance our understanding of mechanisms involved in anastrozole action, all of which have significant clinical implications. For our GWAS, we used 2 independent studies with 2 different but related AI response phenotypes, estrogen suppression and BCFI, and we identified 2 SNPs in the *CSMD1* gene that were associated with 1 of these 2 phenotypes (Figure 1, B and C). However, only rs6990851, and not rs6981827, was eQTL for *CSMD1*. These 2 SNPs have very low linkage disequilibrium. Previous studies have provided evidence that biochemical and genetic manipulations of motif-adjacent sequences can influence transcription factor activity (20, 33). Here, we show that *CSMD1* regulation is estrogen dependent and only the rs6990851 is SNP dependent, while rs6981827 is not. The rs6990851 SNP is in a region that has EREs within 500 bp of the SNP. We demonstrated that the rs6990851 SNP modifies ERα binding to an ERE in the *CSMD1* gene, leading to altered *CSMD1* and *CYP19* expression in an anastrozole-dependent fashion (Figure 2G). Mechanistically, CSMD1 regulated *CYP19A1* gene transcription by acting as a scaffolding protein for SMAD3 and TGF-βR (Figure 3, E and G, and Supplemental Figure 5). The variant rs6990851 SNP allele that increased *CSMD1* expression also increased sensitivity to anastrozole, but not letrozole or exemestane (Figure 3). This result strongly suggested potential differences in mechanisms of action between anastrozole and the other 2 AIs, and this was also confirmed in our previous finding (18). We did not observe an SNP-drug interaction in the MA.27 trial, and the SNP showed a protective effect regardless of the treatment arm, exemestane versus anastrozole. Based on the SNP-dependent drug effects on *CSMD1* and *CYP19* in LCLs, we concluded that the variant rs6990851 SNP results in lower expression of *CSMD1* and, in turn, *CYP19* in the presence of androstenedione, leading to slow cell proliferation and better prognosis. The addition of exemestane or letrozole did not change the *CSMD1* and *CYP19* expression patterns compared with androstenedione treatment (Figure 2, C and D), suggesting that the protective effect of the SNP in the exemestane-treated arm might be mainly due to slow proliferation caused by low *CYP19* expression in subjects carrying the variant SNP. However, treatment with anastrozole in subjects carrying the variant SNP genotype upregulated *CSMD1* and *CYP19*, resulting in increased drug target for AIs. Therefore, we observed a more sensitive phenotype of anastrozole in LCLs containing the variant *CSMD1* rs6990851 SNP (Figure 3A). Therefore, the SNP was a protective SNP with outcomes in MA.27, regardless of the treatment arm. However, based on our results, the mechanisms underlying these association may be different between anastrozole and the other AIs.

The identification of anastrozole and SNP genotype–dependent regulation of gene expression (Figure 2, B–G, and Figure 3) could be explained by a potentially novel mechanism of anastrozole action: anastrozole binds to ERα resulting in ERα degradation (18). At the transcriptional level, anastrozole-dependent transcripts significantly overlapped with E2, but also displayed marked differences from E2, indicating a unique function for anastrozole-ERα gene transcription regulation (Figure 4D). The differences between anastrozole and E2-driven transcriptional programs may be due to differential expression of coregulators that dictate specific ERα-DNA binding and transcriptional activity. Like tamoxifen (34), it is also possible that anastrozole might exert different functions through ERα, either as an agonist or antagonist in a tissue context–dependent manner, a process that is also dependent on the receptor-DNA complex interacting with different coregulators (35). Additional work to decipher these hypotheses is ongoing.

Finally, the effect of anastrozole on ERα protein degradation is potentiated by E2 (Figure 4B). In a short-term randomized study, anastrozole treatment significantly reduced mean ER expression from baseline in breast cancer patients (36). The downregulation of cellular ERα protein occurred without a reduction in ESR1 mRNA, consistent with our findings. Taking advantage of this mechanism, we tested the combination of anastrozole plus E2 as a potential therapeutic strategy, especially in AI- or fulvestrant-resistant breast cancer. We showed that anastrozole plus E2 inhibits proliferation of AI- and fulvestrant-resistant breast cancer cells in addition to its effect in hormone therapy–naive breast cancer cells (Figure 5 and Figure 7). Furthermore, the same results were also observed in breast cancer PDX–derived organoids (Figure 6, A and B). Hence, the combination might offer a potential alternative therapeutic strategy against refractory ER^+^ breast cancer. E2 is being used in the clinic to treat breast cancer with doses as high as 30 mg/d (37). The E2 effect on tumor inhibition has also been observed in tamoxifen-resistant breast cancer in mice, where estrogen can reverse tamoxifen resistance (38). Therefore, the combination of anastrozole plus E2 might be a feasible therapeutic option that could be tested in the future. In our study, we also showed that the combination of anastrozole and E2 was superior to E2 alone (Supplemental Figure 6E), further suggesting that E2 potentiates the effect of anastrozole on ERα degradation. The development of CDK4/6 inhibitors has changed the therapeutic management of HR^+^ MBC. Palbociclib, ribociclib, and abemaciclib are approved in combination with an AI or fulvestrant for HR^+^ MBC (39, 40). Abemaciclib is also approved as a monotherapy for pretreated patients (41). Key questions in the field include whether all patients with HR^+^ MBC should receive a CDK4/6 inhibitor up front and what the mechanism may be of clinical resistance. Patients who progressed on CDK4/6 inhibitor plus AI are currently offered additional therapies such as fulvestrant as a single agent, but the PFS with fulvestrant in the post-CDK4/6 setting is unclear. Therefore, there is a great need to identify additional therapies to improve outcome. Our observations here, combination of anastrozole and E2, may offer additional therapeutic strategies to further improve outcomes of HR^+^ MBC.

Taking advantage of the results of 2 GWAS with related but different phenotypes associated with AI action, we have identified a *CSMD1* SNP as a predicative marker for AI response. Furthermore, the cumulative effect of anastrozole plus E2 on ERα degradation might provide an alternative therapy for ER-positive patients, especially those who are already resistant to hormonal therapy. The differences in E2- and anastrozole-dependent transcription regulation should help us better understand mechanisms of anastrozole resistance and could help identify additional drug targets or treatment options to overcome AI resistance.

## Methods

### GWAS of estrogen response in M3 cohort.

For the GWAS of estrogen suppression to anastrozole therapy, we used samples from the M3 AI Pharmacogenomics Study (19). To ensure that all included patients were compliant with therapy, we included only patients who had detectable levels of either anastrozole or one of its metabolites. Therefore, we excluded 12 patients who had a discordant change in direction between their estrone and E2 concentrations after AI treatment. We also excluded 8 additional patients whose preanastrozole and on-anastrozole E2 concentrations were either undetectable or zero. Our final analysis sample size was 624 patients. To assess SNP effects on estrogen response, 3 phenotypes were analyzed: (a) absolute change in E1; (b) absolute change in E2; and (c) absolute change in the sum of E1 and E2. A van der Waerden transformation was applied to address the skewed Gaussian distributions. Linear regression models were used with additive SNP effects and the following covariates: age, BMI, the first 6 eigenvectors from a principal components analysis, and either baseline E1, E2, or the sum of E1 and E2. Q-Q plots were used to assess adherence of the resultant *P* values to the null distribution. Common genes having SNPs reaching GWAS significance were retained for further analyses. All analyses were performed using R statistical computing software (v3.0.2) and PLINK (v1.07) (7).

### Survival analysis in the MA.27 cohort.

To determine if the SNP was associated with BCFI, the nonparametric log-rank test was initially performed, with each SNP coded as a categorical variable as 0, 1, or 2 copies of the minor allele. SNPs found to be associated with BCFI were then forwarded to a Cox proportional hazards model where SNPs were modeled as a continuous dosage variable. In both cases, the null hypothesis of no association between SNP alleles and BCFI were being tested. Satisfaction of the assumption of proportional hazards was tested after fitting the Cox model in R (version 3.0.2) followed by the Grambsch and Therneau test. To select clinical variables that might confound the relationship between SNP and BCFI, univariate Cox regression models were fitted with drug usage, chemotherapy usage, tumor stage, age, BMI, and PR status separately. For all continuous variables, the linearity assumption was tested, and if violated, that variable was modeled using restricted cubic splines. Variables found to have a significant association (*P* < 0.05) with BCFI were included in a forward stepwise manner to build a multivariate Cox model with SNP. These analyses were performed in the full cohort and also separately within each treatment arm. To determine if the SNP modified the effect of the treatment on BCFI, Cox models were fitted with interaction terms between SNPs and treatment arm. Similarly, interactions between the SNPs and chemotherapy use, progesterone receptor status, and tumor stage status were tested. All analyses were done in R (version 3.0.2).

We performed Fisher’s exact tests to examine the imbalances between North American patients who were included in this GWAS and those who were not. Stepwise selection method was used to evaluate additional clinical variables associated with a breast cancer recurrence event. We employed a stratified genome-wide Cox-proportional hazards model using significant stratification factors with control for additional covariates including treatment arm, cohort, race, ER/PR status, tumor stage, Eastern Cooperative Oncology Group (ECOG) performance score, and bisphosphonate use. All the analyses were run using the R statistical computing package, PLINK, and SAS (SAS Institute) (7).

### RNA-seq analysis and normalization.

T47D cells were treated with vehicle, E2 (0.1 nM), anastrozole (10 nM), or a combination of E2 and anastrozole for 24 hours. RNA-seq was performed and analyzed as described in the Supplemental Methods. The RNA-seq data are publicly available from NCBI Gene Expression Omnibus (http://www.ncbi.nlm.nih.gov/geo) under SuperSeries accession no. GSE150683.

### Organoid derivation, 3D cell culture, and viability assay.

ER+ PDXs from the Breast Cancer Genome Guided Therapy Study (BEAUTY) were generated according to a previously described protocol (28). Tumors were injected s.c. into 6- to 8-week-old female nonobese diabetic/severe combined immunodeficient NOD/SCID/IL-2γ receptor–null (NSG) mice (The Jackson Laboratory). Tumor cells from 2 breast cancer PDXs were isolated using the human Tumor Dissociation Kit (Miltenyi Biotec). Briefly, tumors were minced and then transferred into the gentle MACS C Tube and run through the 7C_h_TDK3 program according to manufacturer’s protocol. The tubes were then centrifuged (2000 *g* for 5 minutes at room temperature) to collect the sample material. Samples were resuspended and applied to a MACS SmartStrainer (70 μm). A Mouse Cell Depletion Kit (Miltenyi Biotec) was used to enrich human cells. Specifically, the cell pellet was suspended in buffer, 20 μL of the mouse cell depletion cocktail was added, and the mixtures were incubated for 15 minutes at 4°C. Then, magnetic separation with LS Columns was performed to collect human cells. Organoids were cultured in 96-well low-binding NanoCulture plate (Organogenix) in DMEM supplemented with 10% FCS, 1% glutamax, 1% sodium pyruvate, nonessential amino acids, and 1% penicillin-streptomycin (Invitrogen) at 37°C, 5% CO_2_. Details of the organoid viability assay are described in the Supplemental Methods.

### Statistics.

For cell survival, cell proliferation, gene expression, and quantifications, data are represented as the mean ± SEM of 3 independent experiments. Unless otherwise described, 2-way ANOVA was performed to test group difference. Then post hoc analysis was carried out to check if specific groups are significantly different or similar. Tukey’s honest significant differences (HSD; R function: TukeyHSD()), which is essentially a modified *t* test corrected for multiple comparisons, was applied in this analysis. Statistical significance level is *P* < 0.05.

### Study approval.

M3 study was reviewed and approved by local IRBs at all participating institutions (Mayo Clinic IRB, M.D. Anderson Cancer Center IRB, and Memorial Sloan Kettering Cancer Center IRB). Written informed consent was obtained from each patient. MA.27 study was approved by local IRBs in accordance with assurances filed with, and approved by, the Department of Health and Human Services. All animal studies were reviewed and approved by the Mayo Clinic IACUC.

## Author contributions

Conception and design were contributed by JC, JNI, and LW. Development of methodology were contributed by JC, TD, LW, JNI, and RMW. Acquisition of data was contributed by JC, JNI, AUB, MER, MJE, PEG, LES, MPG, and BG. Analysis and interpretation of data (e.g., statistical analysis, biostatistics, computational analysis) were contributed by JC, TD, KRK, EEC, JN, HL, and MGB. Writing, review, and/or revision of the manuscript were contributed by JC, JNI, LW, and TD. Administrative, technical, or material support was contributed by JC, TD, EEC, and MGB. Study supervision was contributed by JC, JNI, LW, and RMW.

## Supplementary Material

Supplemental data

Supplemental Table 2

## Version 1 07/23/2020

In-Press Preview

## Version 2 08/20/2020

Electronic publication
