# Supplementary material for: Pharmacogenomics of aromatase inhibitors in postmenopausal breast cancer and additional mechanisms of anastrozole action
Source: JCI Insight. 2020 Aug 20;5(16):e137571. doi: 10.1172/jci.insight.137571 (PMC7455128; doi:10.1172/jci.insight.137571)
Supplement: Supplemental data [file jciinsight-5-137571-s214.pdf]

Supplemental Materials

Pharmacogenomics of aromatase inhibitors in postmenopausal breast cancer and additional mechanisms of anastrozole action

Junmei Cairns[1], James N. Ingle [2], Tanda M. Dudenkov[1], Krishna R. Kalari[3], Erin E. Carlson[3], Jie Na[3], Aman U. Buzdar[4], Mark E. Robson[5], Matthew J. Ellis[6], Paul E. Goss[7], Lois E. Shepherd[8], Barbara Goodnature[9], Matthew P. Goetz[2], Richard M. Weinshilboum[1], Hu Li[1], Mehrab Ghanat Bari[1], Liewei Wang [1#]

Materials

Anastrozole, letrozole, exemestane (Selleckchem, Houston, TX), and MG132 (Sigma-Aldrich, St. Louis, MO) were dissolved in DMSO (Sigma-Aldrich) as 10 mM stocks. 4-androstene-3, 17-dione (Steraloids Inc., Newport, RI), E2 and 4-hyroxy-tamoxifen (4-OH TAM) (Sigma-Aldrich) were dissolved in 100% ethanol for further use. *CSMD1* plasmid was purchased from Origene (Rockville, MD). *CYP19* specific CRISPR/Cas9 Knockout (KO) Plasmid and control plasmid were from Santa Cruz (Dallas, TX).

M3 source of patients

The M3 study enrolled 830 postmenopausal women with resected early-stage breast cancer at the Mayo Clinic (Rochester,MN; Jacksonville, FL; and Scottsdale, AZ), M.D. Anderson Cancer Center (Houston, TX), and Memorial Sloan-Kettering Cancer Center (New York, NY). Eligibility criteria included postmenopausal status; stage I, II, or III breast cancer according to the American Joint Committee on Cancer (AJCC) Staging Manual (Sixth Edition); tumor that was ER and/or progesterone receptor (PR) positive; and planned treatment with the aromatase inhibitor anastrozole at the clinically approved dose of 1 mg per day. Patients could have already received tamoxifen, but prior exposure to other endocrine therapy was not permitted. None of the patients were receiving hormone replacement therapy at the time of the study. Two weeks or less before the initiation of anastrozole therapy, a blood sample was obtained for the isolation of DNA and for the assay of pretreatment plasma estrogens concentrations. This study was reviewed and approved by local institutional review boards at all participating institutions. Written informed consent was obtained from each patient.

Hormone assays and genotyping of the M3 study

Details of the Mayo/M.D. Anderson/Memorial Sloan Kettering (M3) hormone assays were described previously (1). Briefly within two weeks prior to starting anastrozole, a blood sample was obtained for the acquisition of DNA and for pre-treatment E2 and E1 measurements using gas chromatography–tandem mass spectrometry (2) with lower limits of quantitation in this study of: E2, 0.625 pg/mL; E1, 1.56 pg/mL. A second blood draw for E2 and E1 measurements was scheduled for at least four weeks after initiation of anastrozole. DNA from the subjects was genotyped for over 600,000 SNPs by the RIKEN Center for Integrative Medical Science

(Yokohama, Japan) with the Illumina Human610-Quad platform. SNPs with minor allele frequencies (MAFs) <0.01 and/or Hardy–Weinberg equilibrium p values <1.0E−06 were excluded from the analyses. Approximately 8 million SNPs were imputed using reference populations from the 2010 release of the 1000 Genomes Project. Reference SNPs with MAF values <0.005 were removed prior to imputation performed using BEAGLE v3.3.1 (3). Imputed SNPs with dosage r2 values <0.3 and/or MAF values <0.01 were excluded from analyses.

MA.27 source of patients

MA.27, a cooperative-group, multicenter, multinational, randomized, open-label phase 3 trial of exemestane versus anastrozole (4), includes 7576 postmenopausal women with resected stage I-III ERα and/or PgR positive breast cancer. Patients were randomly assigned as follows: 3789 to 5 years of exemestane (25 mg daily) and 3787 to 5 years of anastrozole (1 mg daily). 5221 of 6827 North American patients' blood samples were collected with consent for genetic testing. This research was performed after approval by local institutional review boards in accordance with assurances filed with, and approved by, the Department of Health and Human Services. The primary outcome in our pharmacogenomics studies with MA.27 patients was the STEEP endpoint of Breast Cancer-Free Interval BCFI (5).

MA. 27 genotyping, imputation, and quality control

Three cohorts of patients from MA.27 were genotyped by the RIKEN Center for Integrative Medical Sciences. Cohort 1 involved 888 patients who were genotyped with the Illumina Human610-Quad BeadChip platform (Illumina, San Diego, CA) (6), and 876 samples were available for inclusion in this GWAS. Cohort 2 involved 877 patients genotyped utilizing the Illumina Human OmniExpress platform (7). Cohort 3 involved the remaining 2973 patients from MA.27 with DNA and consent. Genotypes were determined utilizing the Illumina Human OmniExpressExome platform. 2929 samples from Cohort 3 were available for inclusion in this GWAS. The quality control measures for cohorts 1, 2 and 3 have been published (6-8).

Combined Cohort: After removing related samples and merging with available clinical data, a total of 4658 samples were available, including 869 from Cohort 1, 881 from Cohort 2, and 2908 from Cohort 3. Among the 4658 samples, 252 were from patients with breast events.

Cell lines

Human ER+ breast cancer cell lines ZR-75-1 and T47D were obtained from American Type Culture Collection (ATCC, Manassus, VA) in 2014 and the identities of all cell lines were confirmed by the medical genome facility at Mayo Clinic Center (Rochester MN) using short tandem repeat profiling upon receipt. The ER positive breast cancer cell line MCF7/AC1, stably overexpressing CYP19A1 was a generous gift from Angela H. Brodie, Ph. D. (University of Maryland, Baltimore, MD). The cells were authenticated in 2015 by Genetica DNA Laboratories (Cincinnati, OH) using a StemElite ID system that uses short tandem repeat genotyping. A letrozole resistant derivative AC1-LetR was obtained by continuous cultivation of MCF7/AC1 cells in the continuous presence of letrozole (9). The anastrozole resistant MCF7/Ana^{R}-2 and fulvestrant resistant MCF7/164R-7 cell lines were obtained from the European Collection of Authenticated Cell Cultures (ECACC, Salisbury, UK). The MCF7/Ana^{R}-2 cell line was established from MCF7 cells grown in medium with 10% newborn calf serum (NCS) and 10^{-7} M testosterone. A culture of MCF7 cells were treated with 10^{-7} M anastrozole for one week, and seeded in serial dilutions in 24-well plates (10). Single colonies were transferred to new wells and gradually expanded in medium with anastrozole. After ~2–3 months, the isolated colonies gave rise to anastrozole-resistant cell lines, which could be grown in anastrozole-containing medium. The MCF-7 cell line was authenticated in January 2014 by DNA profiling using short tandem repeat loci and found to be matching the genetic profile reported for the MCF7 cell line. The MCF7/164R-7 cell line has been established from a clone of MCF7/S0.5 cells surviving long term growth with the pure steroidal antiestrogen fulvestrant in 100 nM concentration (11). The MCF7/164R-7 cells are also resistant to the pure steroidal antiestrogen fulvestrant. Human differentiated adipocytes were obtained from Cell Applications, Inc. (San Diego, California).

Cell Culture

ZR-75-1 and T47D cells were cultured in RPMI1640 medium with 10% (vol/vol) fetal bovine serum (FBS) (Life Technologies, Carlsbad, CA). MCF7/AC1 cells were cultured in phenol-red free IMEM (Life Technologies, Carlsbad, CA) with 10% FBS and 600 ug/ml Geneticin (Life Technologies, Carlsbad, CA). AC1-LetR cells were cultured in phenol-red free IMEM with 5% Charcoal/Dextran Treated Fetal Bovine Serum (CSS), 600 ug/ml Geneticin, and 1% Pen Strep (P/S). MCF7/AnaR-2 cells were cultured in phenol-red free DMEM/F12 medium supplemented

with 10% newborn calf serum (NCS), 2.5 mM Glutamax (Life Technologies, Carlsbad, CA), 6 ng/µl insulin (Life Technologies, Carlsbad, CA), 0.1µM testosterone, and 0.1 µM anastrozole. MCF7/164R-7 cells were cultured in phenol-red free DMEM/F12 supplemented with 1% FCS, 2.5 mM Glutamax, 6 ng/ml insulin, and 100 nM fulvestrant.

Lymphoblastoid Cell Lines (LCLs) Culture

Five LCLs with CSMD1 WT SNPs and five LCLs with *CSMD1* variant SNPs were identified. Before E2 or androstenedione treatment, ~ 2×10^7 cells from each LCL were cultured in RPMI 1640 medium containing 5% (v/v) charcoal stripped FBS (Invitrogen, Carlsbad, CA) for 24 h, followed with RPMI 1640 medium without FBS for additional 24 h. Each LCL was plated into 12 well plates with RPMI 1640 medium containing 0, 0.001, 0.01, 0.1 and 1 nM E2 or 0, 0.2, 2, and 20 nM androstenedione. After 24 h treatment, increasing concentrations of anastrozole, letrozole, or exemestane were added to the LCLs at concentrations of 0, 1, 10, 100, and 1000 nM. After an additional 24 h, all LCLs were collected for further protein and RNA extraction.

Gene set enrichment analysis

The biological information and attribution embedded in the treatment-associated gene set were analyzed using a web-based integrated data mining system, WebGestalt. Biochemical pathways and functions linked to the resveratrol gene set were specifically queried and navigated by the KEGG pathway enrichment analysis tool in WebGestalt (12). The gene set enrichment analysis searches for combinations of features that show a significant difference in means between two classes that is not an artifact or data noise. Significance in the identified gene sets is confirmed by replacing chosen significant features (i.e., gene sets) with other genes chosen at random, followed by testing and re-analyzing to see whether the significant difference still persists. The validated significant gene sets were then organized based on the KEGG biochemical pathways in a KEGG Table. The top 10 pathways with an adjusted P-value less than 0.01 were selected.

Quantitative real-time PCR assay (qRT-PCR)

QRT-PCR assays were performed for measuring gene expression using Power SYBR® Green RNA-to-CT 1-Step Kit (Life Technologies, Grand Island, NY) and QuantiTect (QIAGEN, Germantown, MD) or PrimeTime (IDT, Inc., Coralville, Iowa) pre-designed qPCR primers.

RNA was extracted using the QIAGEN RNeasy kit (Germantown, MD). RNA was measured by NanoDrops300 (Thermo fisher, Rockford, IL). QRT-PCR reactions were prepared as per the manufacturer's protocol. Samples were run using StepOnePlus real-time PCR system (ABI).

Chromatin Immunoprecipitation (ChIP) assays

ChIP assays were performed using EpiTect ChIP OneDay kit (Qiagen). Approximately 2×10^7 LCLs per every sample (different SNP genotypes with E2 or E2 plus 4-OH TAM treatment groups; androstenedione or androstenedione plus AI) were collected for the ChIP assay. The details for the assay have been described (8). ChIP results were analyzed by qRT-PCR. Primers for SNP rs6990851 and the first ERE: forward primer 5'- AGTGCCACACTGAGCGTTTA-3', reverse primer 5'- CCTTGCTCATCTTCCATGCT-3'; primers for SNP rs6990851 and the second ERE: forward primer 5'- AGTGCCACACTGAGCGTTTA-3', reverse primer 5'- TGCTCATCTTCCATGCTTCA-3'.

siRNA and cDNA construct overexpression

Specific short interfering RNAs (siRNAs) that targeted *SF-1* or *SMAD3* and non-targeting siRNA controls (Dharmacon, Lafayette, CO) were transfected into T47D, ZR75-1 and human adipocyte cells using Lipofectamine RNAiMAX Reagent (Thermo Fisher Scientific, Waltham, MA). *CSMD1* plasmids (Origene, Rockville, MD) were transfected into T47D, ZR75-1, adipocytes, MCF7/AC1, and AC1-LetR cells using Lipofectamine 2000 Reagent (Thermo Fisher Scientific, Waltham, MA). Total RNA was extracted 48 hours after transfection for RNA quantification. Whole cell lysates were collected 48 hours after transfection for Western blots.

Cell proliferation assays

For cell proliferation after E2 or AI treatment, cells were starved in culture medium with 5% charcoal stripped FBS for 24 hours followed by various treatments. Specifically, 3000 cells were plated per well in 96 well plates in triplicate, followed by different treatments depending on the experiments: 10 nM E2, 100 nM AI, 10 nM E2 plus 100 nM AI. BrdU Cell Proliferation Assay kit (Cell Signaling, Danvers, MA) were used to determine the cell viability daily, following the manufacturer's instructions.

Cell survival assays

Cytotoxicity for human tumor cell lines was determined by quantification of DNA content using CyQUANT Direct Cell Proliferation Assay kit (#C35012, Invitrogen) following the manufacturer's instructions. Specifically, cells were seeded onto 96-well plates in triplicate at each drug concentration. After 24 hours, cells were treated with 10 µL of AIs at final concentrations of 0, 0.0001, 0.001, 0.01, 0.1, 1, 10, 100, 1000, and 10000 nM. After incubation for an additional 72 hours, 100 µL of CyQUANT assay solution was added, and plates were incubated at 37°C for one hour, and then read in a Safire2 plate reader with filters appropriate for 480 nm excitation and 520 nm emission.

Western blotting

Cells were washed with cold PBS and were lysed in cold NETN buffer (100 mM NaCl, 20 mM Tris·HCl, 0.5 mM EDTA, NP-40) with Protease inhibitor cocktail (Roche, Indianapolis, IN) and phosphatase inhibitor (PhosSTOP EASY pack, Roche). Cell lysates were diluted with SDS loading buffer (SDS, glycerol, bromic acid, 1 M Tris·HCl) and boiled, centrifuged at 10,000 rpm and stored at -20 °C. Equal amounts of protein were subjected to electrophoresis in 4-15% TGX SDS gels (Bio-Rad, Hercules, CA) and were transferred to PVDF membranes. Membranes were blocked in TBS with 5% BSA and 0.1% Tween-20 and then incubated overnight at 4 °C with the following primary antibodies: ERα (EPR4097) (108398, Abcam), SMAD3 (C67H9) (9523, Cell Signaling Technology), p-SMAD3 (SAB4503781, Sigma-Aldrich), TGFβR1 (3712, Cell Signaling Technology), CSMD1 (EPR8656(2)) (166908, Abcam), CYP19A1 (D5Q2Y) (14528, Cell Signaling Technology), vinculin (hVIN-1) (V9131, Sigma-Aldrich) and β-actin antibodies (AC-15) (A1978, Sigma-Aldrich). Membranes were washed with TBS-T (TBS with 0.1% Tween-20) and then incubated with HRP-conjugated anti-mouse IgG (7076, Cell Signaling Technology) or HRP-conjugated anti-rabbit IgG (7074, Cell Signaling Technology) for 1 hour at room temperature. All blots were visualized with Supersignal WestPico chemiluminescent ECL kit (Thermo Fisher) and blue X-ray films (Phenix, Candler, NC).

Co-Immunoprecipitation Assay

Cells were lysed with NETN buffer (20 mM Tris–HCl, pH 8.0, 100 mM NaCl, 1 mM EDTA, 0.5% Nonidet P-40) containing 50 mM β-glycerophosphate, 10 mM NaF, and 1 mg/ml each of pepstatin A and aprotinin on ice for 25 min. After centrifugation, cell lysates were incubated

with 2 μg antibody and protein A sepharose beads (Amersham Biosciences) for 3 h at 4°C. After centrifugation, the beads were then washed four times with NETN buffer and immunoprecipitates were separated by SDS–PAGE. Immunoblotting was performed following standard procedures.

Generation of *CYP19* Knockout cells by CRISPR/Cas9 technology

CYP19 CRISPR/Cas9 KO plasmid and HDR plasmid were obtained from Santa Cruz Biotechnology. 2 x 10^5 T47D cells were seeded in 6-well tissue culture plate, and were transfected with 1 μg of *CYP19* CRISPR/Cas9 KO plasmid and 1 μg of HDR plasmid using Lipofectamine 2000. 72 hrs later, puromycin selection was performed for the following 4 weeks to isolate *CYP19* CRISPR/Cas9 KO single clones. *CYP19* mRNA and protein were analyzed to confirm the knockout efficiency.

Luciferase activity assay

Transcription activity of ERα was measured using the dual luciferase assay with the Cignal ERE Reporter Assay Kit (Qiagen). *CYP19* CRISPR knockout and control T47D cells were cultured in charcoal stripped serum medium for 48 hours and then switched to serum free medium in 96-well plates for 2 days. Cells were then transfected with either ERE reporter (inducible ERα-responsive GFP reporter), negative control (GFP reporter construct with GFP expression controlled by a minimal promoter), or Positive control (constitutively expressing GFP construct) constructs using the Lipofectamine 2000 transfection reagent. After 16-24 hours of transfection, cells were treated with E2, AI, or E2 plus AI for 48 hrs. Luciferase assay was performed using the Dual-Luciferase Reporter Assay System from Promega following the manufacturer's protocol.

RNA-Seq analysis and normalization

RNA was prepared from cells using the TRIzol (Life Technologies) extraction kit. Genomic DNA was removed using the Ambion DNA-free kit. NuGEN Encore reagents were used for library preparation of total RNA samples. One microgram of total RNA input was used for each sample. The libraries were sequenced on an Illumina HiSeq 2000 sequencing system using 100-bp single-ended reads. After removing the poor-quality bases from FASTQ files for the whole

transcriptome sequencing, paired-end reads are aligned by reads that were aligned to the human reference genome UCSC hg19 with Tophat 2.0.14 and the bowtie 2.2.6 aligner option. Transcript abundance was estimated using a count-based method with htseq-count. Gene counts were used as input for TMM (Trimmed Mean of M values) normalization of the R package edgeR 3.18.1, and the normalized counts were transformed to log2-counts per million (logCPM) by applying voom from the R package limma to account for higher variability at low expression levels. Genes with zero read counts across all samples were removed.

Organoid viability assay

Viability of organoids was measured using the luminescent CellTiter-Glo Viability assay (Promega). Organoids were seeded (10,000 organoids/well) in 96-well NanoCulture plate in medium with 5% charcoal stripped FBS (Invitrogen) for 24 hrs, followed with culture medium without FBS for additional 24 hrs. Organoids were then treated with 10 µL of AIs or fulvestrant at final concentrations of 0, 0.0001, 0.001, 0.01, 0.1, 1, 10, 100, 1000, and 10000 nM in triplicate. For the AIs plus E2 treatment, the E2 concentrations were 0, 0.1, 1, 10, and 100 nM. After incubation for an additional 72 hours, 100 µL of CellTiter-Glo reagent was added to each well, followed by mixing contents for 2 minutes on an orbital shaker to induce cell lysis. Plates were incubated at 37°C for additional one hour, and the luminescent signal was measured in a Safire2 plate reader.

References

1. Ingle JN, Buzdar AU, Schaid DJ, Goetz MP, Batzler A, Robson ME, Northfelt DW, Olson JE, Perez EA, Desta Z, Weintraub RA, Williard CV, Flockhart DA, Weinshilboum RM. Variation in anastrozole metabolism and pharmacodynamics in women with early breast cancer. Cancer Res. 2010 Apr 15; 70 (8):3278-86 Epub 2010 Mar 30 PMID: 20354183 PMCID: 2855746 DOI: 10.1158/0008-5472.CAN-09-3024.
2. Prokai-Tatrai K, Bonds D, Prokai L. Simultaneous Measurement of 17beta-Estradiol, 17alpha-Estradiol and Estrone by GC-Isotope Dilution MS/MS. Chromatographia **2010**;71(3-4):311-5 doi 10.1365/s10337-009-1441-0.

3. Browning SR, Browning BL. Rapid and accurate haplotype phasing and missing-data inference for whole-genome association studies by use of localized haplotype clustering. Am J Hum Genet **2007**;81(5):1084-97 doi 10.1086/521987.
4. Goss PE, Ingle JN, Pritchard KI, Ellis MJ, Sledge GW, Budd GT*, et al.* Exemestane versus anastrozole in postmenopausal women with early breast cancer: NCIC CTG MA.27--a randomized controlled phase III trial. J Clin Oncol **2013**;31(11):1398-404 doi 10.1200/JCO.2012.44.7805.
5. Hudis CA, Barlow WE, Costantino JP, Gray RJ, Pritchard KI, Chapman JA*, et al.* Proposal for standardized definitions for efficacy end points in adjuvant breast cancer trials: the STEEP system. J Clin Oncol **2007**;25(15):2127-32 doi 10.1200/JCO.2006.10.3523.
6. Ingle JN, Schaid DJ, Goss PE, Liu M, Mushiroda T, Chapman JA*, et al.* Genome-wide associations and functional genomic studies of musculoskeletal adverse events in women receiving aromatase inhibitors. J Clin Oncol **2010**;28(31):4674-82 doi 10.1200/JCO.2010.28.5064.
7. Liu M, Goss PE, Ingle JN, Kubo M, Furukawa Y, Batzler A*, et al.* Aromatase inhibitor-associated bone fractures: a case-cohort GWAS and functional genomics. Mol Endocrinol **2014**;28(10):1740-51 doi 10.1210/me.2014-1147.
8. Ingle JN, Xie F, Ellis MJ, Goss PE, Shepherd LE, Chapman JW*, et al.* Genetic Polymorphisms in the Long Noncoding RNA MIR2052HG Offer a Pharmacogenomic Basis for the Response of Breast Cancer Patients to Aromatase Inhibitor Therapy. Cancer Res **2016**;76(23):7012-23 doi 10.1158/0008-5472.CAN-16-1371.
9. Macedo LF, Sabnis G, Brodie A. Aromatase inhibitors and breast cancer. Ann N Y Acad Sci **2009**;1155:162-73 doi 10.1111/j.1749-6632.2008.03689.x.
10. Thewes V, Simon R, Hlevnjak M, Schlotter M, Schroeter P, Schmidt K*, et al.* The branched-chain amino acid transaminase 1 sustains growth of antiestrogen-resistant and ERalpha-negative breast cancer. Oncogene **2017**;36(29):4124-34 doi 10.1038/onc.2017.32.
11. Lykkesfeldt AE, Larsen SS, Briand P. Human breast cancer cell lines resistant to pure anti-estrogens are sensitive to tamoxifen treatment. Int J Cancer **1995**;61(4):529-34.

12. Wang J, Duncan D, Shi Z, Zhang B. WEB-based GEne SeT AnaLysis Toolkit (WebGestalt): update 2013. Nucleic Acids Res **2013**;41(Web Server issue):W77-83 doi 10.1093/nar/gkt439.

Supplemental Figures and Figure Legends

Supplemental Figure 1. (**A**) Flow chart of the M3 GWAS pipeline. (**B**) Manhattan plot of M3 GWAS results. (**C**) The rs6990851 SNP cis-eQTL with *CSMD1* in GTEX.

Supplemental Figure 1

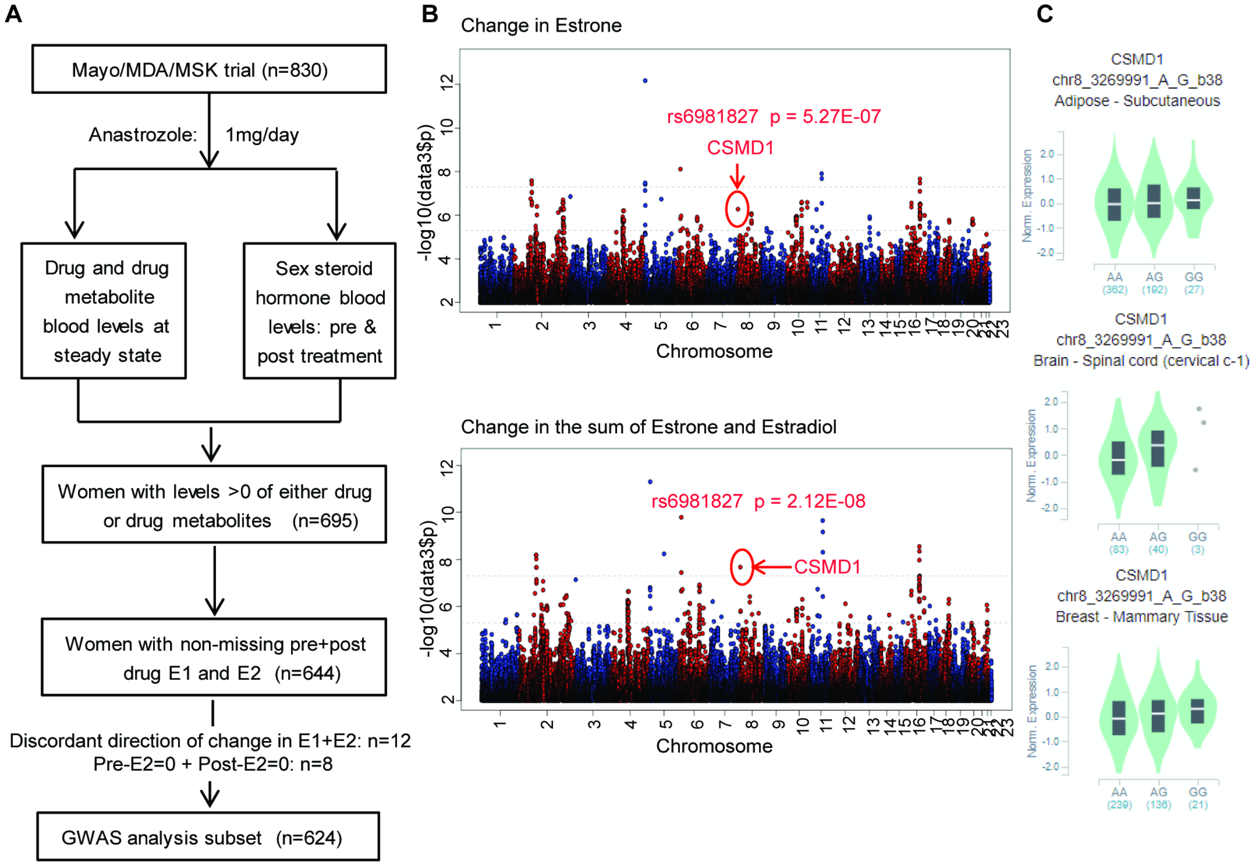

Supplemental Figure 2. *CSMD1* mRNA expression after exposure to 0.1 nM estradiol (E2) for various times up to 48 hours in human ZR75-1 and T47D cells. Error bars represent the SEM of three independent experiments (relative expression). *p < 0.05; ** p< 0.01. Statistical test: 2-way ANOVA.

Supplemental Figure 2

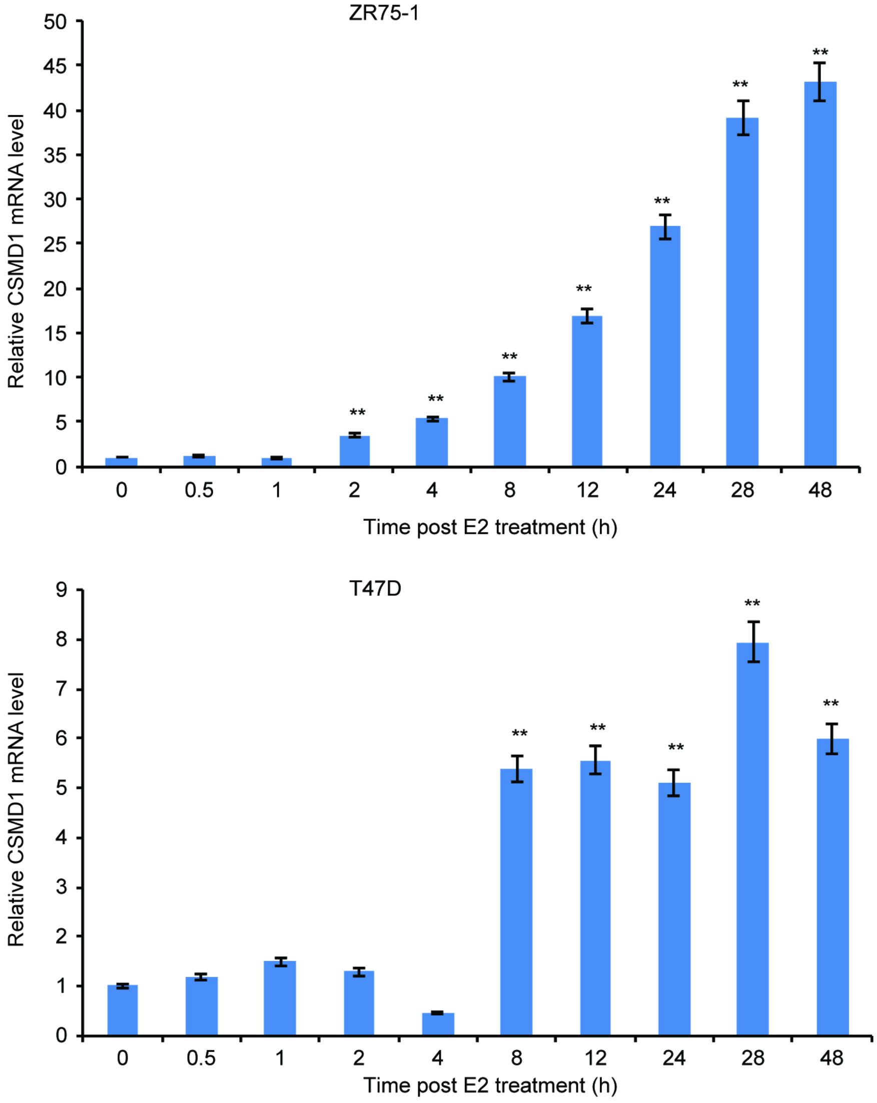

Supplemental Figure 3. ERα ChIP assay shows *CSMD1* SNP effect on ERα binding to ERE 291 bp downstream from rs6990851 in the presentence of androstenedione (A) with or without AIs. Error bars represent the SEM of three independent experiments. *p < 0.05; ** p< 0.01. Statistical test: 2-way ANOVA.

Supplemental Figure 3

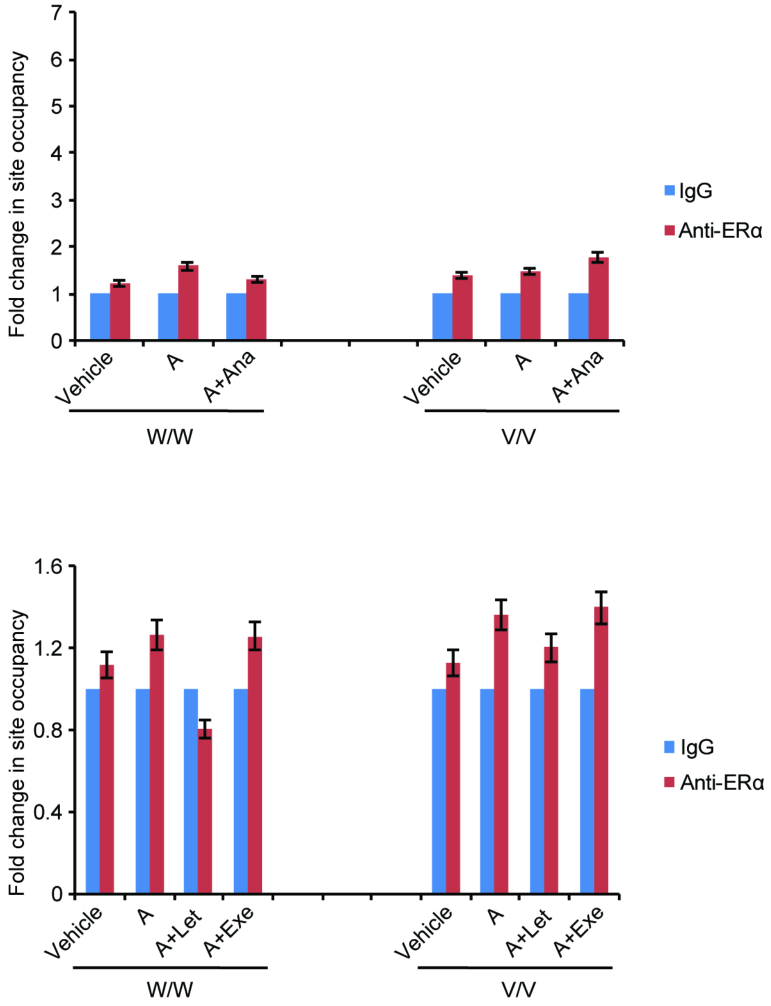

Supplemental Figure 4. *CSMD1* effects on AI response in breast cancer cells. AI sensitivity after overexpressing *CSMD1* or empty vector (EV) in T47D and ZR75-1 cells. Error bars represent the SEM of three independent experiments. *p < 0.05; ** p< 0.01. Statistical test: 2-way ANOVA plus Tukey.

Supplemental Figure 4

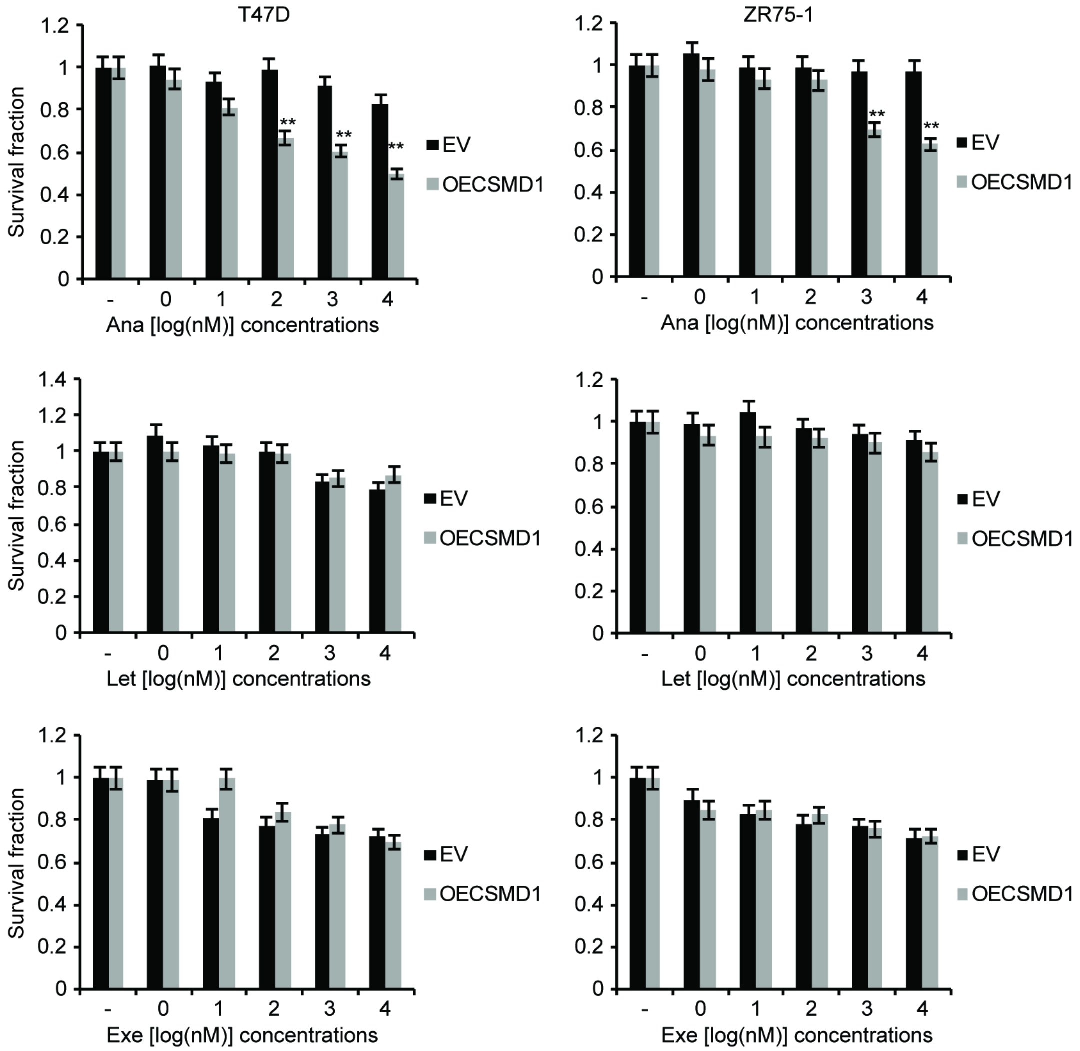

Supplemental Figure 5. (**A-D**) *CSMD1* regulates *CYP19A1* expression through *SF-1* and *SMAD3*. T47D, ZR75-1 and human adipocyte were transfected with indicated siRNA or plasmid. 48 h later, mRNA and cell lysate were harvested for qRT-PCR and western blot. Error bars represent the SEM of three independent experiments. *p < 0.05; ** p< 0.01. Statistical test: 2-way ANOVA plus Tukey.

Supplemental Figure 5

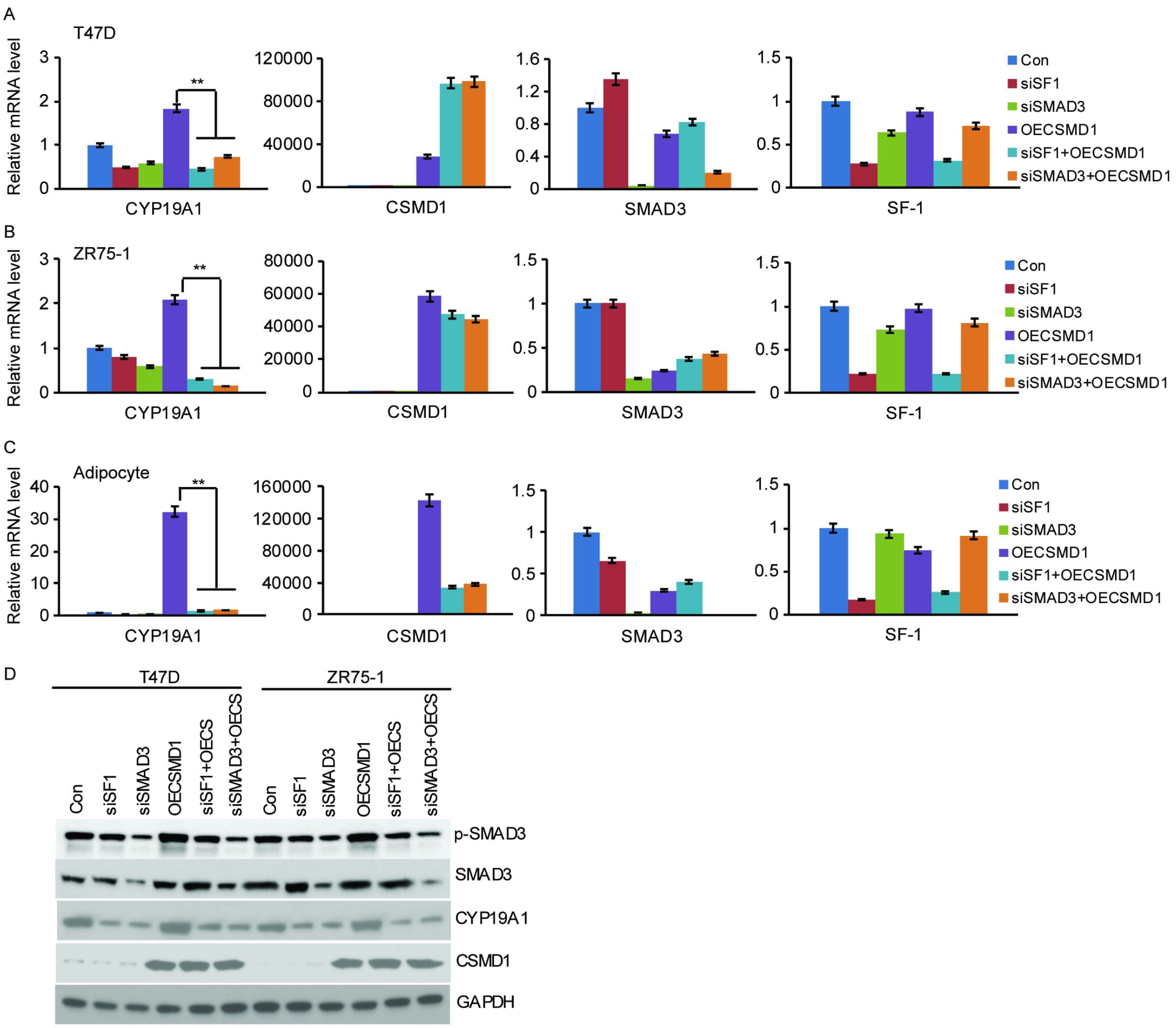

Supplemental Figure 6. (**A**) Differentially expressed genes in response to anastrozole with or without E2 in T47D cells (FDR<0.05). (**B**) Validation of selected genes using qRT-PCR. (**C**) E2 dose response in *CYP19* KO T47D, AC1-LetR, and MCF7/AnaR cell lines. (**D**) Response of fulvestrant-resistant breast cancer cells to E2 plus AIs treatment. Error bars represent the SEM of three independent experiments. *p < 0.05; ** p< 0.01. Statistical test: 2-way ANOVA plus Tukey.

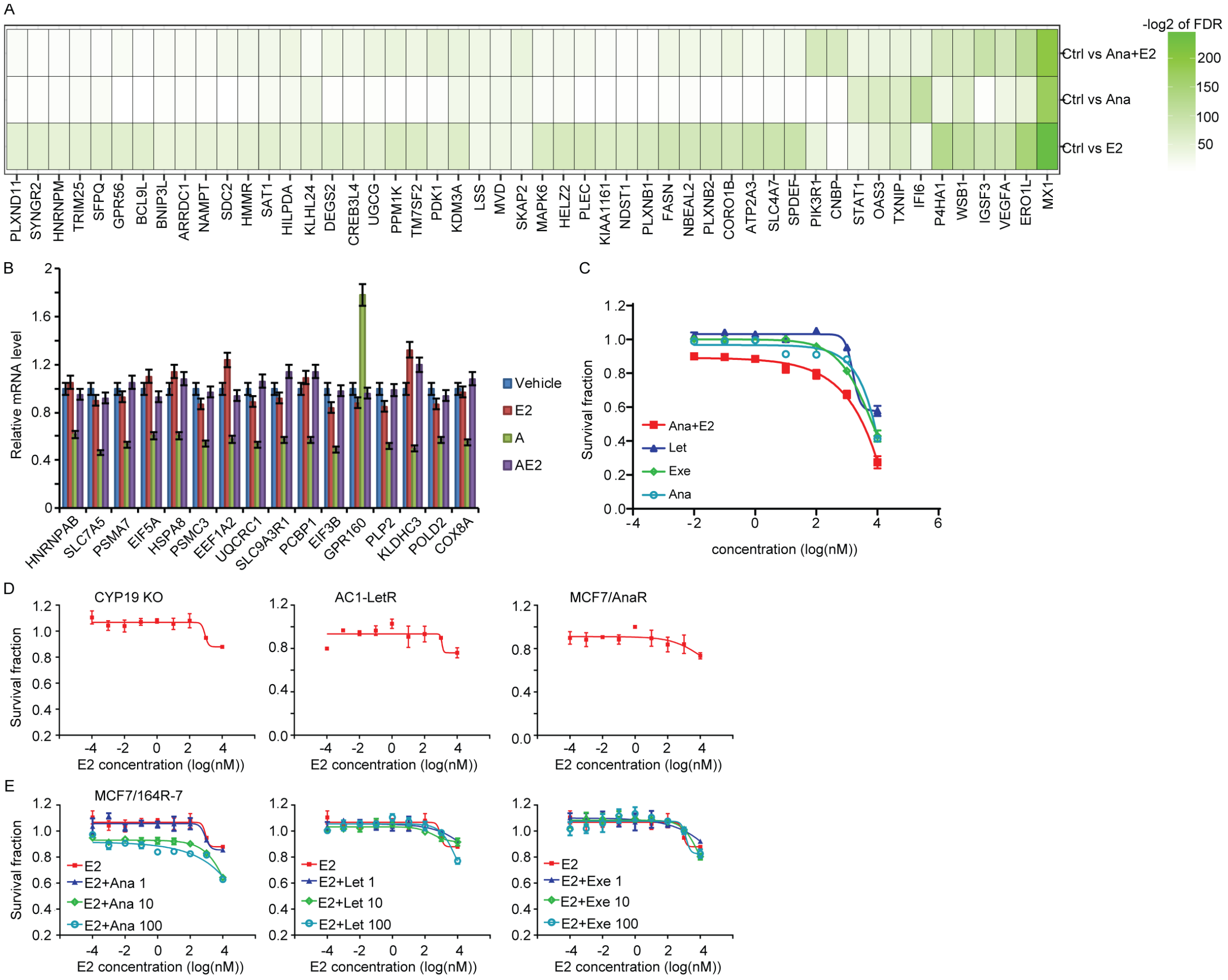

Supplemental Table 1: Overlapped SNPs associated with changes in estrogens with p<5E-8 in M3 study.

SNP	CHR	BP	A1	MAF	BETA	SE	P	Type	Gene	DistfromSNP
rs2449598	11	83372420	A	0.04808	-0.4897	0.07589	2.23E-10	G	DLG2	0
rs1437153	16	64444588	G	0.0618	-0.4028	0.0668	2.82E-09	I	CDH11	149443
rs6981827	8	3075038	C	0.0502	-0.5978	0.1053	2.12E-08	I	CSMD1	0

Supplemental Table 3: Pathways enriched in genes altered by anastrozole and E2 in T47D cells as determined by Web[illegible]

Anastrozole

**PathwayName**	**#Gene**	**EntrezGene**
Metabolic pathways	44	7357 6480 7086 353 26227 10682 23475 27430 1595 3705 55902 1337 191 1[illegible]
Steroid biosynthesis	6	6713 7108 1595 4047 1717 10682
DNA replication	7	4173 5981 5426 4171 4172 2237 5425
Insulin signaling pathway	9	207 208 5295 3099 2194 6720 2872 5499 5573
Toxoplasmosis	9	5163 207 23118 208 5295 3312 3949 6772 598
RNA transport	10	1981 23225 23511 1975 1917 1974 3646 8662 51068 10460
Protein processing in endoplasmic reticulum	10	7184 5589 30001 3309 3312 821 3998 823 55666 7324
VEGF signaling pathway	7	5829 207 7422 3315 10725 208 5295
Regulation of actin cytoskeleton	11	54961 2934 5829 4659 5216 5295 60 5499 5217 3985 1072
Pancreatic cancer	7	7046 598 207 7422 208 5295 6772

E2

**PathwayName**	**#Gene**	**EntrezGene**
Metabolic pathways	97	7357 4191 3074 537 4711 2752 23475 3422 2806 230 7385 2805 8733 10768[illegible] 353 5297 51477 26227 27430 4723 5298 5211 6307 1337 10606 8833 123099[illegible] 2821 1717 132 9791 3712
Protein processing in endoplasmic reticulum	31	7184 80331 1388 5589 267 3326 3309 30001 11253 51726 84447 821 7324 5[illegible]
Pathways in cancer	42	9134 2033 7184 3728 6256 2064 54205 1026 598 329 25 1387 1399 5925 57[illegible]
Focal adhesion	31	5058 5829 207 4659 7094 208 394 5295 5908 2064 2316 7422 5501 10627 3[illegible]
Regulation of actin cytoskeleton	30	5058 54961 23191 5829 4659 8826 5295 4627 10787 26999 5217 5501 1062[illegible]
Hepatitis C	23	207 5163 208 6256 5295 3949 3646 1026 6773 10401 5515 3716 5518 5601 [illegible]
RNA transport	24	23511 1975 1974 6612 3646 9631 22985 8021 11260 8667 9883 8761 7341 7[illegible]
Insulin signaling pathway	22	207 208 5295 10211 2194 23265 867 5501 805 5792 1399 5601 3099 6720 5[illegible]
Spliceosome	21	11338 6628 6431 3183 22985 9416 6426 23450 22827 84991 22938 6427 10[illegible]

Tight junction	21	57530 207 208 1457 4627 7525 10627 84612 5515 27134 3993 5728 5518 55

**Anastrozole plus E2**		
**PathwayName**	#Gene	EntrezGene
Metabolic pathways	51	2548 204 3636 2180 4329 7357 1718 2683 427 4942 9453 2132 10682 23475 2821 1717 5214 1737
Pathways in cancer	22	7184 5914 7170 3326 5295 2064 7046 54205 598 1026 7422 1399 3716 5728
Glycolysis / Gluconeogenesis	10	2597 5230 55902 230 226 3099 2821 2023 5214 1737
Pentose phosphate pathway	7	51071 2821 6888 230 5214 226 5226
Hepatitis C	12	3716 5163 5295 6772 3646 1364 8503 1026 440275 4940 975 5594
Steroid biosynthesis	6	6713 7108 4047 1717 1718 10682
Insulin signaling pathway	12	1399 6464 5295 3099 2194 6720 2872 8503 23265 5499 5594 31
Prostate cancer	10	148327 7184 5728 3326 5295 367 2064 8503 1026 5594
Pancreatic cancer	9	8503 2064 7046 598 3716 7422 5594 5295 6772
Chronic myeloid leukemia	9	8503 1399 7046 598 861 1026 6464 5594 5295
